# Extracellular vesicles from recombinant cell factories improve the activity and efficacy of enzymes defective in lysosomal storage disorders

**DOI:** 10.1002/jev2.12058

**Published:** 2021-03-12

**Authors:** Joaquin Seras‐Franzoso, Zamira V. Díaz‐Riascos, José Luis Corchero, Patricia González, Natalia García‐Aranda, Mònica Mandaña, Roger Riera, Ana Boullosa, Sandra Mancilla, Alba Grayston, Marc Moltó‐Abad, Elena Garcia‐Fruitós, Rosa Mendoza, Guillem Pintos‐Morell, Lorenzo Albertazzi, Anna Rosell, Josefina Casas, Antonio Villaverde, Simó Schwartz, Ibane Abasolo

**Affiliations:** ^1^ Drug Delivery & Targeting CIBBIM‐Nanomedicine Vall d'Hebron Institute of Research Universitat Autònoma de Barcelona Barcelona Spain; ^2^ Networking Research Center on Bioengineering Biomaterials and Nanomedicine (CIBER‐BBN) Barcelona Spain; ^3^ Institut de Biotecnologia i de Biomedicina (IBB) and Department of Genetics and Microbiology Universitat Autònoma de Barcelona (UAB) Bellaterra Barcelona Spain; ^4^ Functional Validation & Preclinical Research (FVPR) CIBBIM‐Nanomedicine Vall d'Hebron Institut de Recerca (VHIR) Universitat Autònoma de Barcelona (UAB) Barcelona Spain; ^5^ Nanoscopy for Nanomedicine Group Institute for Bioengineering of Catalonia (IBEC) Barcelona Spain; ^6^ Neurovascular Research Laboratory Vall d'Hebron Institut de Recerca (VHIR) Universitat Autònoma de Barcelona (UAB) Barcelona Spain; ^7^ Division of Rare Diseases Reference Center for Hereditary Metabolic Disorders (CSUR, XUEC, MetabERN, and CIBER‐ER) Vall d'Hebron University Hospital Barcelona Spain; ^8^ RUBAM, Biological Chemistry Institute of Advanced Chemistry of Catalonia (IQAC‐CSIC) Barcelona Spain; ^9^ Networking Research Center on Hepatic and Digestive Diseases (CIBEREHD) Barcelona Spain

**Keywords:** alpha‐galactosidase A, drug delivery, enzyme replacement therapy, Fabry disease, lysosomal storage disorders, N‐sulfoglucosamine sulfohydrolase, Sanfilippo syndrome

## Abstract

In the present study the use of extracellular vesicles (EVs) as vehicles for therapeutic enzymes in lysosomal storage disorders was explored. EVs were isolated from mammalian cells overexpressing alpha‐galactosidase A (GLA) or N‐sulfoglucosamine sulfohydrolase (SGSH) enzymes, defective in Fabry and Sanfilippo A diseases, respectively. Direct purification of EVs from cell supernatants was found to be a simple and efficient method to obtain highly active GLA and SGSH proteins, even after EV lyophilization. Likewise, EVs carrying GLA (EV‐GLA) were rapidly uptaken and reached the lysosomes in cellular models of Fabry disease, restoring lysosomal functionality much more efficiently than the recombinant enzyme in clinical use. In vivo, EVs were well tolerated and distributed among all main organs, including the brain. DiR‐labelled EVs were localized in brain parenchyma 1 h after intra‐arterial (internal carotid artery) or intravenous (tail vein) administrations. Moreover, a single intravenous administration of EV‐GLA was able to reduce globotriaosylceramide (Gb3) substrate levels in clinically relevant tissues, such kidneys and brain. Overall, our results demonstrate that EVs from cells overexpressing lysosomal enzymes act as natural protein delivery systems, improving the activity and the efficacy of the recombinant proteins and facilitating their access to organs neglected by conventional enzyme replacement therapies.

**ABBREVIATIONS**
BSA: bovine serum albumin
DHFR: dihydrofolate reductase
ERT: enzyme replacement therapy
EVs: extracellular vesicles
GAGs: glycosaminoglycans
Gb3: globotriaosylceramide
GLA: alpha galactosidase A
i.a.: intra‐arterial
i.v.: intravenous
LAMP1: Lysosome‐associated membrane protein 1
LSDs: lysosomal storage disorders
MTX: methotrexate hydrate
PEI: polyethylenimine
SGSH: N‐sulfoglucosamine sulfohydrolase
SN: supernatants

## 1 INTRODUCTION

The use of recombinant proteins to treat a wide variety of clinical indications, including cancer, autoimmune and genetic diseases, is still a challenge (Leader et al., 2008). Lysosomal storage disorders (LSDs) are congenital rare diseases caused by the lack or malfunction of proteins involved in lysosomal biogenesis and activity. There are more than 70 different LSDs, among them, Gaucher, Fabry, Pompe and Sanfilippo syndromes (Platt et al., 2018). All LSDs share the accumulation in the lysosomal compartment of complex molecules such as glycosaminoglycans, glycoproteins and sphingolipids, rendering in most cases severe clinical manifestations (Platt et al., 2018). Patient symptomatology depends on the disease and the particular mutation but often, LSDs derive in a systemic illness affecting multiple organs, including the central nervous system (CNS), liver, kidneys, heart and the musculoskeletal system. Regrettably, LSDs have been historically neglected by the pharmaceutical industry. Their individual low incidence has discouraged active search for treatment due to the high difficulties to organize clinical trials and poor expected post‐commercialization profits. Nevertheless, in the last years, enzyme replacement therapies (ERTs) based on the systemic administration of a functional version of the defective enzyme, have gained clinical relevance in LSD healthcare. Hence, ERTs using recombinant proteins are currently available for 10 LSDs, (Concolino et al., 2018) including Fabry disease. Unfortunately, systemically administered enzymes are not able to reach the brain parenchyma, leaving without effective treatment LSD patients with CNS affectation (Solomon & Muro, 2017).

Fabry disease is an X‐linked disease characterized by the accumulation of globotriaosylceramide (Gb3) and other glycosphingolipids in cellular lysosomes, caused by a deficient activity of the alpha‐galactosidase A (GLA) enzyme, secondary to pathogenic genetic variants of the *GLA* gene. Endothelial cells are one of the most affected cell types. Without proper treatment, alterations in the micro‐ and macro‐vasculature in different organs eventually lead to a multisystemic failure and early death (Rombach et al., 2010). Two products, agalsidase beta (Fabrazyme), and agalsidase alfa (Replagal), are currently approved for ERT in Fabry disease (Beck et al., 2004; Eng et al., 2001). Other treatment approaches based on chaperones, (Hughes et al., 2017) substrate reduction (Guérard et al., 2018) and gene therapy (Huang et al., 2017; Medin et al., 2019) have been either approved recently for Fabry disease (migalastat), or are under study, but with limited efficacy and/or applicability, so far. Conversely, ERT has shown to delay the disease progression in Fabry patients with an early diagnosis (Mehta et al., 2009). Nevertheless, the success of ERT in Fabry disease and other LSDs is often limited by the generation of autoantibodies against the exogenous recombinant proteins, low plasma half‐life and poor biodistribution of the enzymes that fail to effectively target organs such kidney, heart, brain and bone, among others (Safary et al., 2018).

In fact, many LSDs have not yet benefited from ERT due to the impossibility of recombinant enzymes to cross the blood brain barrier (BBB). This is the case of Sanfilippo syndrome, caused by four different genetic defects affecting lysosomal heparan sulfate degradation, each one defining a different disease subtype A, B, C and D. In this regard, Sanfilippo A represents 60% of all diagnosed cases and is caused by a deficiency of the heparan N‐sulfatase enzyme, also known as N‐sulfoglucosamine sulfohydrolase (SGSH). As with the other subtypes, the accumulation of partially degraded glycosaminoglycans (GAGs) in the brain causes progressive cognitive decline and severe behavioural disturbances. Although no therapy is still approved for Sanfilippo disease, several clinical trials are being conducted using intrathecally applied recombinant protein, gene therapy and substrate reduction therapy (Gaffke et al., 2018).

In this scenario, the use of nanotechnological approaches increasing the efficiency of recombinant enzymes and targeting them to specific cell types is regarded as a powerful approach to introduce new effective therapies for LSDs (Muro, 2010). Different types of nanoparticles have been tested as vehicles for ERT in Fabry disease including liposomes, (Cabrera et al., 2016) chitosan polyelectrolytes (Giannotti et al., 2016) and polystyrene nanoparticles (Hsu et al., 2014). Moreover, other nanocarriers from a biological origin, such as nanoparticles made of albumin and 30Kc19 protein (Lee et al., 2016) have also been studied at the preclinical level. Unfortunately, none of these synthetic nanoparticles have been able to advance towards clinical application in LSDs.

In parallel, extracellular vesicles (EVs) have been also proposed as delivery vehicles for recombinant proteins (Sutaria et al., 2017). EVs, including microvesicles (100‐1000 nm) and exosomes (30‐150 nm), naturally contain biomolecular cargos such as miRNA, mRNA and proteins from the donor cells. They have been described as fully biocompatible submicron‐sized vehicles with higher in vivo transfection capacity and lower immunogenic responses than other non‐viral platforms (Srivastava et al., 2016). Importantly, EVs have been shown to be fully tunable using genetically modified cells as source. For instance, Alvarez‐Erviti *et al*. (Alvarez‐Erviti et al., 2011) modified dendritic cells to express Lamp2b, a lysosomal membrane protein, fused to the neuron‐specific RVG peptide to target EVs into the brain parenchyma. Furthermore, EVs can be also chemically and mechanically loaded after their production and isolation, allowing the introduction of siRNA, enzymes and chemotherapeutics by sonication, saponification or simple incubation. (Batrakova & Kim, 2015; Sutaria et al., 2017)

Notably, the delivery of a therapeutic biomacromolecule often requires previous production and purification of the soluble form of the biopharmaceutical and its further load into the vehicle. This increases the complexity of the production and the final cost. Therefore, an all‐in‐one process in which the carrier and the therapeutic cargo are produced jointly may provide an optimal strategy to improve the production process and further, the efficacy of protein‐based therapeutics. This is especially relevant in the case of LSDs, in which ERT is often regarded as ideal standard of care.

In this work, we investigate the use of protein loaded EVs from protein recombinant cell factories for the delivery of therapeutic lysosomal enzymes such GLA and SGSH, by purifying EVs precisely from the same cells used to produce the recombinant enzyme. Enzyme loaded EVs retain highly active GLA and SGSH recombinant enzymes, withstand lyophilization, and are fully biocompatible. Moreover, in vitro and in vivo efficacy assays using GLA loaded EVs, have shown clear advantages on the use of EV‐loaded system vs. the naked recombinant enzymes.

## 2 RESULTS AND DISCUSSION

### 2.1 Mammalian cell factories produce EVs with high quantities of GLA and SGSH enzymes

Enzymatic deficiency of GLA and SGSH proteins cause Fabry and Sanfilippo A diseases, two of the LSDs with higher prevalence (Meikle et al., 1999). In this work we aimed at testing the feasibility of EVs as enzyme delivery systems for GLA and SGSH proteins, as a way to improve ERT in these two diseases. Consequently, EVs were isolated from CHO DG44 and HEK293 cells transfected with plasmids coding for GLA and SGSH enzymes, respectively. Stable clones were obtained from CHO DG44 cells for GLA expression, while transfections in HEK293 cells were conducted transiently for SGSH.

**FIGURE 1:**
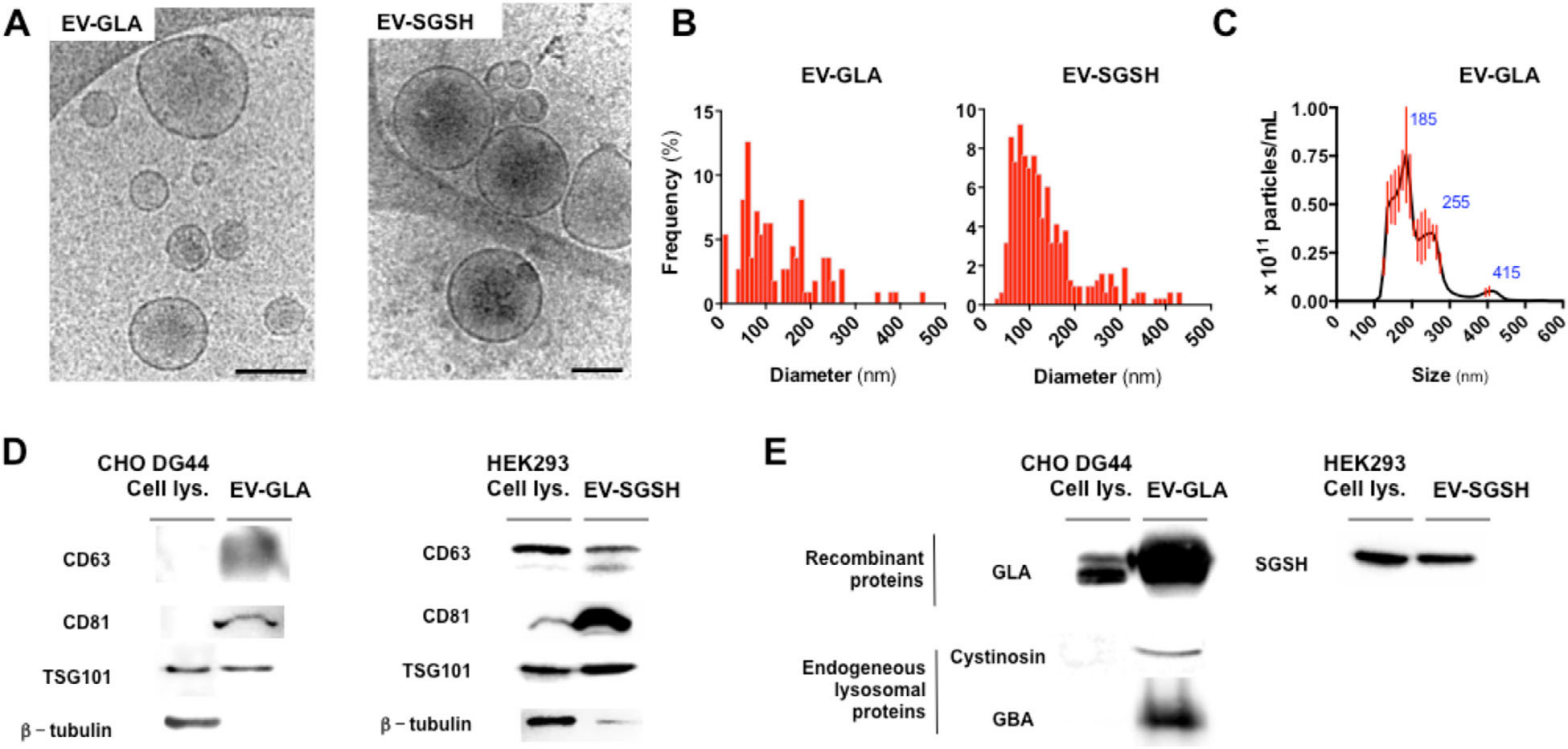
Characterization of EVs containing GLA and SGSH lysosomal enzymes (EV‐GLA and EV‐SGSH). **A)** Characterization by cryoTEM imaging of vesicle morphology. Magnification bar corresponds to 200 nm. **B)** Size distribution of EV‐GLA and EV‐SGSH measured in cryoTEM images. **C)** EV‐GLA size distribution and concentration determination by nanoparticle tracking analysis. Blue values correspond to the size of the main peaks in the histogram. **D)** Molecular characterization of isolated EVs by Western blot of EV markers. Cell lysates and a cytosolic protein (β‐tubulin) were also included as controls. **E)** Lysosomal protein content in EVs by Western blot. *Panel above*, detection of over expressed protein of interest, GLA and SGSH. *Panel below*, detection of additional lysosomal endogenous protein (Cystinosin and GBA) in EV‐GLA. 10 to 20 μg of total protein loaded per lane in both D and E panels

EVs obtained from CHO DG44 and HEK293 cells were purified using charge neutralization‐based precipitation method and later characterized by cryoTEM and Western blot. EVs presented spherical and pseudo‐spherical morphologies that ranged from 30 to 300 nm in diameter by cryoTEM and Nanosight assessment (Figure 1 **A‐C**). Additional cryoTEM images at lower magnification are available in **Figure S1**. Western blot analyses showed very low content of cytosolic β‐tubulin within the vesicles and the presence of exosome markers CD63, CD81‐tetraspanin and TGS101 (Théry et al., 2018) (Figure 1 **D**) indicated that EVs purified from CHO DG44 and HEK293 cell‐factories were enriched in exosomes (50 to 150 nm) and small‐sized microvesicles, with some of large microvesicles and apoptotic bodies (Figure 1 **A**). Such mixed population of vesicles was expected because the size‐range of exosomes partially overlap with other types of EVs and purification protocols currently in use, mainly based on ultracentrifugation and precipitation methodologies, do not render pure populations of different types of EVs (Kowal et al., 2016). Noteworthy, significant amounts of the protein of interest, GLA and SGSH, were found in EV lysates in comparison to whole cells lysates (Figure 1 **D**). Of note, EVs and lysosomes are both originated from the multivesicular endosomes (MVE) (Raposo & Stoorvogel, 2013) and this common origin could explain the high GLA and SGSH enzyme concentration within the EVs. Although other possibilities, such the release of lysosomal proteins in EVs due to autolysosomal dysfunctions, could not be excluded (Goetzl et al., 2015). Notwithstanding, other endogenous lysosomal proteins such as the membrane protein cystinosin and the beta‐glucocerebrosidase (GBA) enzymes, were also found enriched in EV‐GLAs, suggesting EV could be an adequate platform for naturally loading a broader spectrum of lysosomal proteins (Figure 1 **E**).

Regarding the presence of the therapeutic proteins in the EVs, the fact that 4.48% and 10.25% of the total protein in the EVs corresponded to GLA and SGSH respectively when isolated by polymer co‐precipitation method is remarkable (**Figure S2A**). Of note, although EV precipitation is a convenient method for preclinical studies, due to its simplicity and speed, it can also co‐isolate contaminant proteins in their soluble form. In order to characterize the extent of this phenomenon an alternative purification method, namely tangential flow filtration (TFF) (Busatto et al., 2018) was carried out, we detected the production yield dropped a 42% when using TFF method compared to the precipitation kit used in the isolation of EVs for in vitro and in vivo studies. However, we also found that the enzymatic activity within the EVs was the major contributor to the increased activity seen in the original EV‐GLA samples, as demonstrated by the high enzymatic specific activities in the TFF‐purified samples, non‐ significantly distant from the original EV‐GLA (**Figure S2A**). This indicated that the efficacy seen upon EV‐GLA treatment of cell cultures or Fabry animals could be mainly attributed to the effect of the GLA in the EV lumen and not to the GLA co‐purified with the EVs. Since the solely TFF might not remove all the free soluble proteins (Corso et al., 2017), to further confirm the higher performance of GLA loaded into EVs in comparison to its soluble counterpart co‐isolated during EV precipitation, precipitated EV‐GLA were submitted to and additional sucrose cushion purification step (Théry et al., 2006). With this purification method, the relative amount of GLA decreased a 60.5 ± 0.6% in comparison to the original sample (**Figure S2B**). However, enzymatic activity of GLA inside EVs was significantly higher, 6 times more active, than its soluble co‐isolated counterpart (**Figure S2B)**. Regarding SGSH protein the amount of soluble protein co‐purified was higher reaching the 85.9± % of the initial enzyme in the EV‐SGSH precipitated samples. These results were in agreement with the ones observed for TFF purification and ratified the major contribution of EV‐GLA to the efficacy observed in vitro and in vivo. Further, an iodixanol density gradient separation (Lobb et al., 2015) onto EV‐GLA precipitated samples was carried out (**Figure S2C)** and revealed significant levels of GLA protein in different density fractions. Considerable amounts of GLA were found in low density fractions (< 1.06 g/cm^3^), probably corresponding to soluble protein interacting with Low Density Lipoprotein (LDL) particles (Karimi et al., 2018). This phenomenon was likely enhanced by the high concentration of samples subjected to sucrose gradient, at least 1 order of magnitude above those subjected to TFF or sucrose cushion methods. Remarkably, GLA was also found, as expected, at densities corresponding to EVs (1.08 – 1.14 g/cm^3^) that retained a significant part of the enzymatic activity (**Figure S2C**) whilst expressing EV makers (Bobrie et al., 2012).

Overall, considering the above results we found the actual protein loading capacity at 2.96 fg GLA per vesicle. Of note, this loading capability is almost 30 times higher than the one previously reported for alternative lysosomal enzymes in macrophage‐derived EVs (0.1 fg/ EV) (Haney et al., 2019). Altogether these data suggest that protein loading in EVs can be significantly boosted by selecting cell factories optimized for protein production (Geisse & Fux, 2009). Differences between GLA and SGSH protein content in EVs might be related to the fact that transient transfections, used in this case to obtain EV‐SGSH, usually render higher protein yields than stable transfections (Bandaranayake & Almo, 2014). On the other hand, recombinant protein content in EVs was also compared to the amount of soluble protein found in cell supernatants. Interestingly, supernatants from stably transfected CHO cells contained 41.4 ± 7.14 μg of GLA per ml of cell culture, while 0.517± 0.01 μg of GLA in EVs were obtained per ml of cell culture. Nonetheless, even though the content of GLA in EVs is 80‐fold lower than in cell supernatant, it is worth mentioning that EV loading of GLA is considerably higher than the loading of GLA found in artificial liposomes (Cabrera et al., 2016) or previously reported in EVs loaded with alternative lysosomal proteins (Haney et al., 2019).

### 2.2 Recombinant EV‐GLA restores lysosomal GLA enzymatic activity in GLA defective cells

**FIGURE 2:**
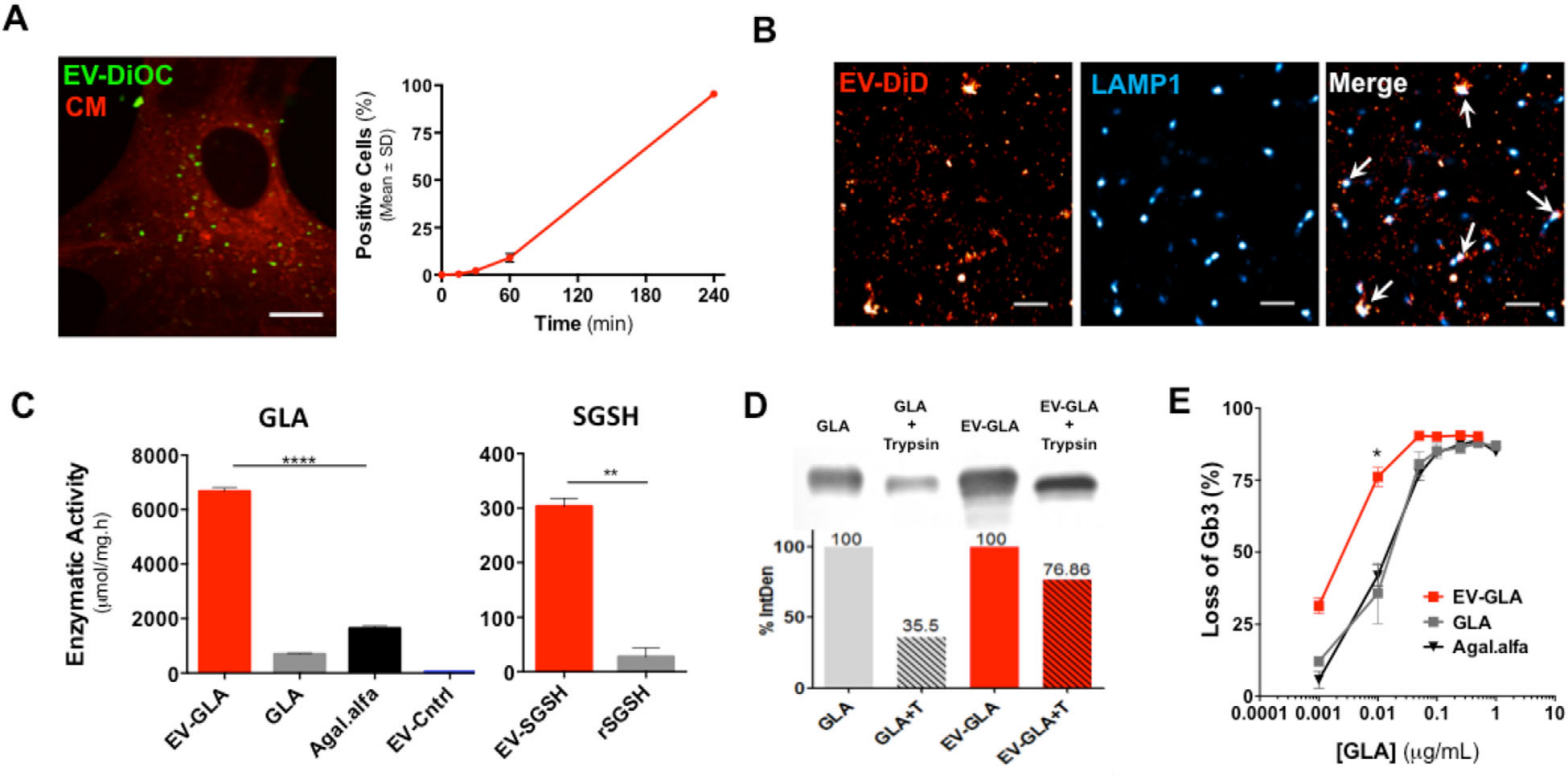
In vitro assessment of EVs as drug delivery systems for lysosomal proteins. **A)** Internalization of EVs by confocal microscopy (4 h incubation) and flow cytometry in primary cultures of mouse aortic endothelial cells (MAEC) derived from Fabry KO mice. EVs were labelled with DiOC (green) and cell membranes were labelled with Cell Mask (red). Magnification bar corresponds to 10 μm. **B)** Inset of HEK293 cytoplasmatic region of a single cell showing colocalization (white arrows) of EV‐GLA labelled with DiD (red) with lysosomal marker LAMP1 (blue) by STORM imaging. Magnification bar corresponds to 1 μm. **C)** Enzymatic activities for alpha‐galactosidase A (left) and heparan sulfatase (right) measured in EVs obtained from cells overexpressing GLA and SGSH proteins, respectively. EVs from non‐transfected CHO cells were also included as controls (EV‐Control). **D)** Protease digestion assay of EV‐GLA (red bars) and their naked GLA counterparts (grey bars). T stands for trypsin treatment. %IntDen refers to the percentage of the integrated density. **E)** Efficacy of EV‐GLA reducing the Gb3 deposits in MAEC primary cultures at different GLA protein concentrations

The capacity of EVs to act as drug delivery system for lysosomal proteins was studied in vitro through the assessment of cell uptake, intracellular trafficking, protease sensitivity and enzymatic activity assays. Fluorescently labelled EVs efficiently internalized in different type of cells, including kidney cells (HEK293T cells, **Figure S3**) and endothelial cells (MAEC, Figure 2 **A**) at a concentration of 2.5  μg EVs/ml (c.a. 125 ng GLA/ml). These later cells were included since endothelial cells are central in the pathophysiology of Fabry disease (Bodary et al., 2007). After short 4 h incubation with fluorescently labelled EVs, nearly 100% of MAEC showed strong fluorescent signal by flow cytometry. Confocal microscopy further confirmed that fluorescently labelled EVs were located inside the cells and not merely attached to the cell membrane. The release of EVs into lysosomes of destiny cells has been extensively described in different cellular models (Do et al., 2019; Hansen et al., 2020; Joshi et al., 2020). In our specific case, EVs preferred cell uptake pathways were further explored by the assessment of diverse internalization inhibitors performance during EV‐GLA uptake process (**Figure S3**). Multiple routes were found involved in the internalization of EV‐GLA as highlighted by the impairment in cell fluorescence intensity after 2 h of EVs incubation in cells pretreated with: dynasore (DYN) 80 μM –inhibition of endocytic vesicle scission from cell membrane–, chlorpromazine (CHP) 20 μM –inhibition of clathrin mediated endocytosis–, nystatin (NYS) 50 μM –inhibition of caveolae mediated endocytosis– and 5‐(N‐ethyl‐N‐isopropyl) amiloride (EIPA) 100 μM –inhibition of macropynocitosis– (**Figure S3D,** left graph). Interestingly, looking at longer EVs incubation times, 4 h, only EIPA maintained the magnitude of the inhibition while the other inhibitors became ineffective (DYN and NYS) or reduced their effect (CHP) (**Figure S3D,** right graph). These results suggested a predominant contribution of clathrin mediated endocytosis and macropynocitosis in our EV‐GLA uptake, in agreement with previous reports (Li et al., 2020).

Further, final intracellular fate of EVs was assessed in HEK293 cells by the co‐localization of EV‐GLA with LAMP1 lysosomal marker by stochastic optical reconstruction microscopy (STORM), a technique that allows the visualization of individual EVs (Nizamudeen et al., 2018). Co‐localization between EVs’ signal and LAMP1 was marginally detected 4 h post administration (**Figure S4**) and clearly observed after overnight incubation, representing the 4.2% ± 1.7% of the total number of EV internalized (Figure 2 **B**). EVs have been shown to enter the cells by different routes, and not all of them necessarily ending into the lysosomal compartment (Mulcahy et al., 2014). According to our results, a significant part of the EVs were uptaken through endocytosis, reaching the lysosome after the endosomal maturation and allowing content release of GLA and the hydrolysis of its substrate. A similar process of cellular uptake has been recently described for EVs containing GBA (Do et al., 2019) and TPP1^35^ lysosomal enzymes.

Once we confirmed that enzyme‐loaded EVs reached the lysosomal compartment, we further explored if GLA and SGSH enzymes kept their activity when delivered in EVs. Remarkably, not only protein quantity but specific enzymatic activity was also significantly increased in GLA and SGSH encapsulated in EVs (Figure 2 **C**). Thus, in EV‐GLA, 10‐fold higher enzymatic activity was detected compared to the activity of soluble GLA from cell supernatants (*P* < 0.0001). Also, 11‐fold increase in activity was found in EV‐SGSH, compared to commercially available recombinant SGSH (rSGSH, *P* = 0.0042). GLA enzymatic activity was also measured in non‐transfected CHO cells, rendering very low but detectable enzymatic activity levels. It is possible that such significant increases in GLA and SGSH activities inside EVs could be due to a higher stability of the enzymes within a more physiological environment, namely acidic pH and a membranous scaffold, as previously described for GLA loaded into artificial lipid structures (Cabrera et al., 2016). Nevertheless, no increments in enzymatic activity have been reported for other lysosomal enzymes loaded in EVs by cell transfection over the cell lysates, namely TPP1, (Haney et al., 2019) HGSNAT (Fedele et al., 2018) and GBA (Do et al., 2019). Therefore, further research is needed to unveil protein conformational quality attributes within EVs and determine whether other enzymes and proteins with catalytic activity could also benefit from EVs encapsulation. In addition, previous studies have suggested that EVs offer natural protection to the enzymes from the action of proteases (Haney et al., 2019). This is also the case for EV‐GLA. As shown in Figure 2 **D**, incubation EV‐GLA with trypsin, a serine protease that will degrade GLA or any protein/peptide having serine residues, resulted in a 25% loss of protein content, whereas such loss was increased to a 65% when naked GLA was trypsin‐treated using the same conditions. The loss of GLA in EV‐GLA samples could be attributed to the presence of free GLA co‐precipitated with the EV‐GLA during the isolation procedure (**Figure S2**)

Overall, cell internalization and crude enzymatic activity assays suggested that EVs are suitable delivery systems for lysosomal enzymes. For further confirmation that EVs could be therapeutically effective, in vitro efficacy assays were conducted in MAEC derived from Fabry KO mice, a very handful in vitro model of the Fabry disease (Shu et al., 2005). Indeed, due to the lack of endogenous alpha‐galactosidase A activity, these cells accumulate high levels of Gb3, the predominant storage product in Fabry patients and the main responsible of the clinical manifestations of the disease (Desnick et al., 2001). In MAEC cells, at 0.001 μg/ml of GLA, EV‐GLA were four to five times more efficacious hydrolyzing Gb3, than soluble GLA (Figure 2 **E,** *P*  = 0.0381). Remarkably, EV‐GLA also overcame the efficacy of agalsidase alfa, the enzyme used in the clinical setting (*P*  = 0.0286, at 0.001 μg/ml of GLA).

### 2.3 EVs withstand lyophilization and are safe for intravenous administration

**FIGURE 3:**
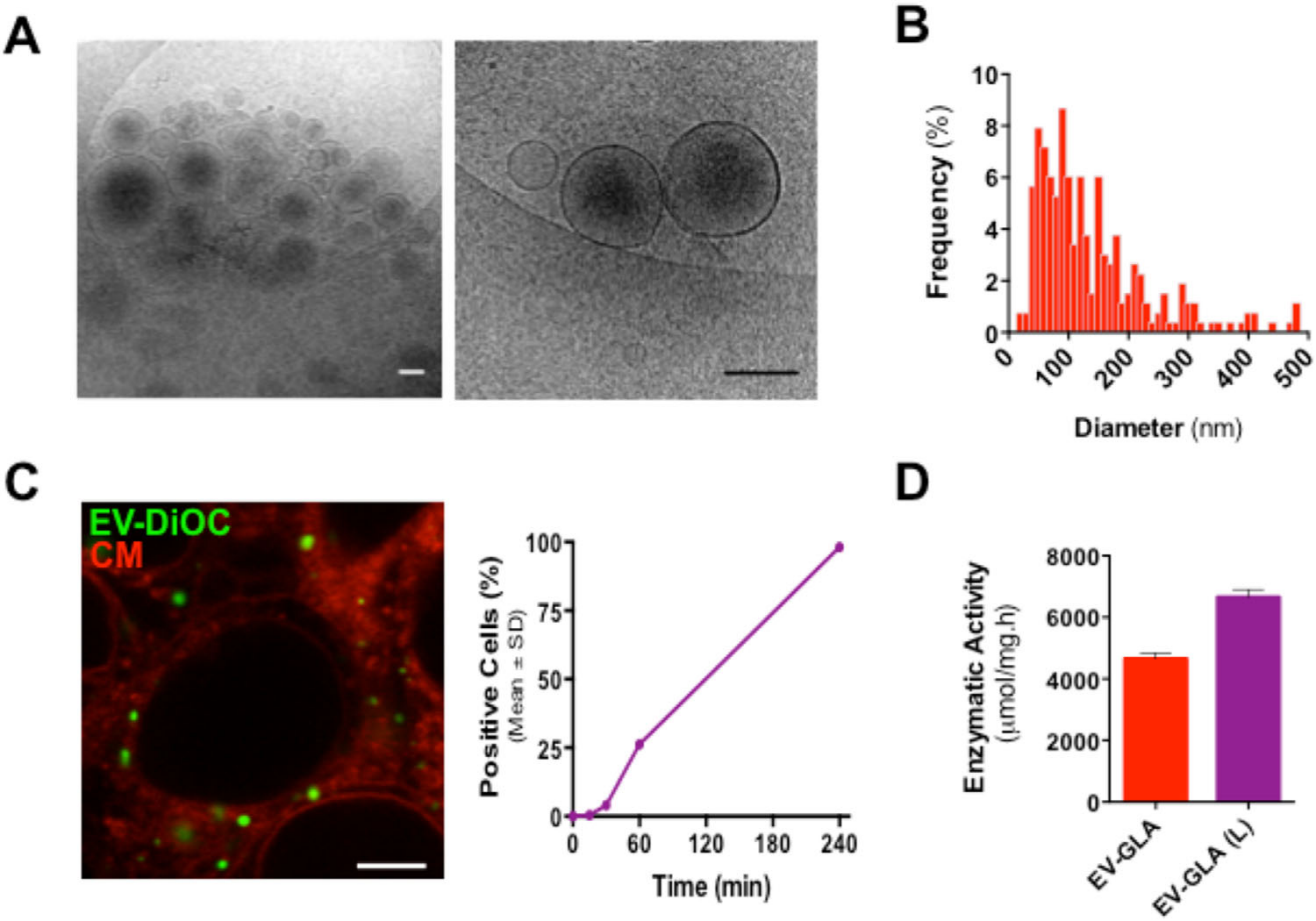
EVs stability after lyophilization. **A)** CryoTEM images of EV‐GLA after lyophilization. Magnification bars correspond to 200 nm. **B)** Size distribution of EV‐GLA post‐lyophilization. **C)** Cell internalization of lyophilized EV by confocal microscopy (left panel) and flow cytometry (right panel) in HEK293 cells. For microscope imaging, EVs were labelled with DiOC (green) and cell membranes stained with Cell Mask (CM, red). DiD‐labelled EV‐GLA were used for cell cytometry. Magnification bar corresponds to 5 μm. **D)** Specific enzymatic activity of GLA in EVs before (red bar) and after lyophilization (purple bar)

Knowing that pharmaceutical development of EVs would require an off‐the‐shelf product with preserved bioactivity, we studied the stability of EVs after lyophilization. Even in the absence of cryopreservants, EV‐GLA withstood lyophilization with minor changes in the vesicle morphology (Figure 3 **A, B**). Lyophilized EVs maintained enzymatic activity values comparable to those of non‐lyophilized EVs and kept the ability to cell‐internalize, showing that lyophilization was not largely affecting EVs’ biofunctionality (Figure 3 **C, D**). Certainly, freeze drying was previously shown to be a useful method to preserve enzymes in EVs (Frank et al., 2018), although sugar additives were thought to be indispensable to prevent EV aggregation and functionality (Bosch et al., 2016).

Because ERT based on EVs would likely relay on the intravenous (i.v.) administration of EV‐GLA, hemocompatibility and safety studies were also conducted (**Figure S5**). Results showed that EV‐GLA did not alter cell proliferation capacity (**Figure S5C**), caused damage to red blood cells nor did they induce any platelet significant aggregation (**Figure S45,B**), which allowed further testing in animal models. Accordingly, C57BL6 mice were administered with 7 i.v. doses of EVs. As expected, none of the animals showed adverse effects as a consequence of the repeated administration of the EVs or enzymes. Moreover, body weights were comparable to control (non‐treated) mice, also suggesting an adequate safety profile (**Figure S5D**). These results are in agreement with previous studies showing the safety of EVs (Fleury et al., 2014; Li et al., 2016; Somiya et al., 2018; Wiklander et al., 2015).

**FIGURE 4:**
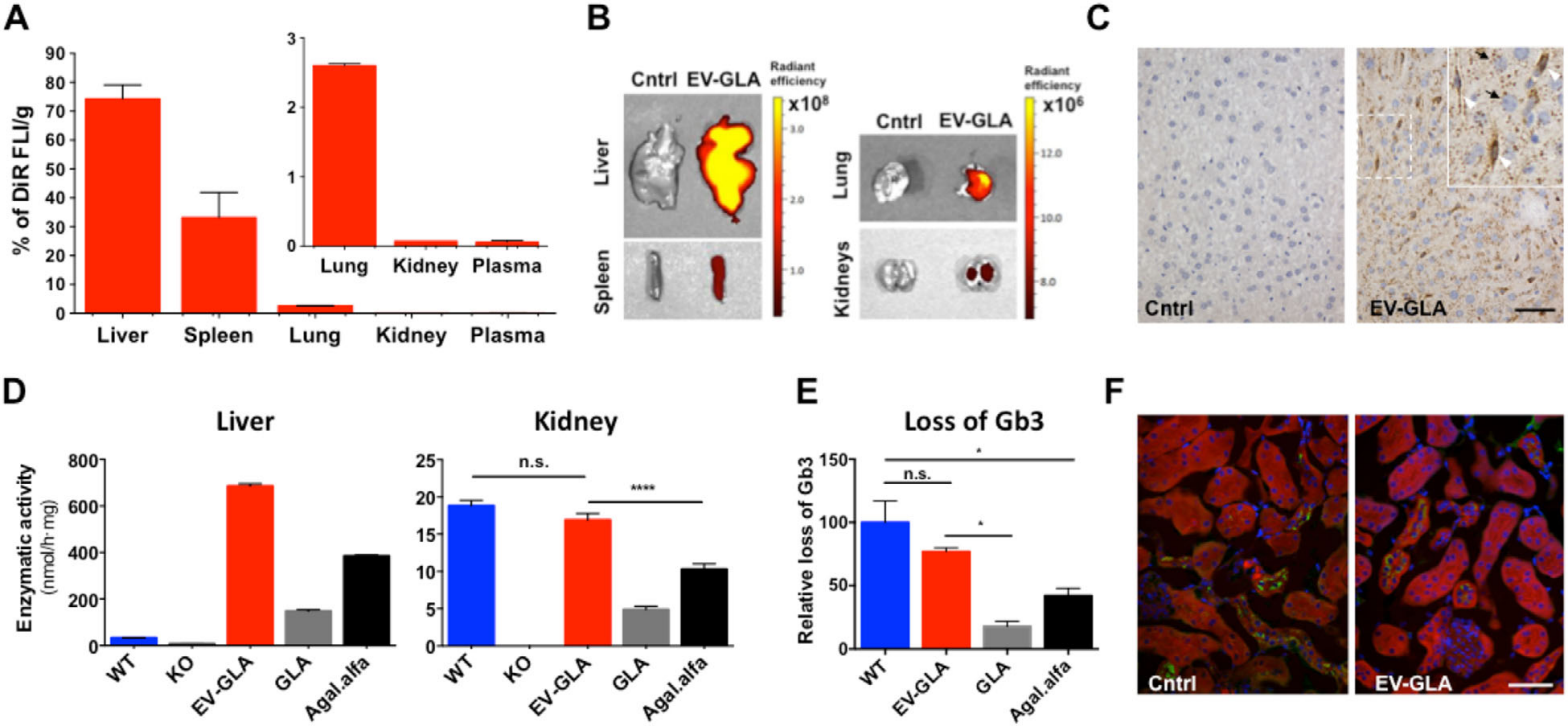
In vivo biodistribution and single‐dose efficacy of EV‐GLA in Fabry KO mice. **A)** Biodistribution of DiR‐labelled EVs in Fabry KO mice 1 h after i.v. administration (100 μg of protein) showed a widespread distribution of the fluorescent signal in different organs. **B)** Ex vivo fluorescence images of the liver and kidneys. **C)** GLA protein detected by immunohistochemistry in liver tissues of GLA KO mice administered with vehicle (Cntrl) or EV‐GLA. Magnification bar corresponds to 50 μm. Inset contains a magnified area to identify hepatocytes (black arrows) and Kupffer cells (withe arrowheads) (**D)** GLA enzymatic activity measurements of mice treated with a single administration of EV‐GLA, free enzyme GLA or agalsidase alfa at 1 mg GLA/kg and euthanized 1 h post‐administration in liver and kidneys. WT animals and non‐treated KO animals were also included in the assay. **E)** Loss of Gb3 in KO mice treated with a single dose of EV‐GLA, free enzyme GLA or agalsidase alfa (1 mg/kg) and euthanized 1 week after. **F)** Immunofluorescence of Gb3 (green signal) in kidneys of KO animals, vehicle‐treated (Cntrl) or receiving one i.v. dose of EV‐GLA (1 mg/kg). Nuclei were stained with DAPI (blue) and cells with rhodamine‐phalloidin (red). Magnification bar corresponds to 40 μm

In addition, biodistribution assays were conducted with DiR‐labelled EV‐GLA Fabry KO mice were sacrificed 1 h post‐administration and tissue samples taken for ex vivo fluorescence imaging. In the first biodistribution assay (Figure 4), EV signal was mainly observed in liver and spleen, while minor but significant amount of fluorescence was also detected in kidneys and lungs (Figure 4 **A,B**). No signal has been observed in the heart, brain, skin and muscles (not shown). These results agree with previous studies on EV biodistribution showing major accumulation in liver and spleen after systemic EV administration (Wiklander et al., 2015). Furthermore, the delivery of the GLA along with the EV was first confirmed by the immunodetection of the GLA protein in livers of Fabry KO mice (Figure 4 **C**). Thus, both, hepatocytes and Kupffer cells from the livers of KO mice treated with EV‐GLA showed strong GLA signal, whereas non‐treated KO mice showed a complete absence of the GLA protein. EV distribution was also confirmed by measuring GLA enzymatic activity in organs of mice administered with 1 mg/kg of GLA protein as EV‐GLA, free GLA or clinically approved agalsidase alfa (Figure 4 **D**). Activity assays were in agreement with the tissue biodistribution of EV‐GLA shown by fluorescence imaging and GLA immunohistochemistry. In our hands, for the same GLA dose, more enzymatic activity was seen in livers and kidneys from animals administered with EV‐GLA compared to those receiving free GLA or agalsidase alfa. In the case of the liver, administration of exogenous GLA, either naked or encapsulated in EV, resulted in a significant increase of enzymatic activity in the organ, far beyond the endogenous activity seen in wild type (WT) animals. This is probably related to the fact that most of the administered dose is accumulated in the liver. Conversely, in kidneys where few EVs arrived according to fluorescence biodistribution (0.377% ± 0.04% of the total fluorescent signal), GLA enzyme delivered by the EVs increased the enzymatic activity up to the normal levels detected in healthy WT animals (no statistically significant differences among groups), while the free GLA or the clinically approved agalsidase alfa only recovered 60% of the activity seen in kidneys of WT mice (*P*  < 0.0001). Hence, for the same GLA administered dose, EVs bring significantly more enzymatic activity to the kidneys’ than non‐encapsulated enzyme. This is probably due to the fact that the delivery of enzyme within EVs allows the combination of three different phenomena: increased intrinsic enzymatic activity, protection against protease degradation, and advantageous differential uptake. In the context of ERT, it is worth noting that the distribution and tissue accumulation of the free GLA are governed by presence of mannose‐6‐phospate receptors (M6PR), which are highly expressed in the liver (Sly et al., 2006). However, as cell internalization of EVs does not depend on the presence of M6PR, other tissues with lower M6PR expression such as kidneys, might particularly benefit from ERT treatments based on EVs. This could be especially relevant in Fabry disease, where most of classic male and some female patients present advanced stages of chronic kidney disease (Sunder‐Plassmann, 2006).

To measure the impact of EV‐GLA treatment in the kidneys, Gb3 levels were determined in KO animals receiving a single dose GLA, EV‐GLA or agalsidase alfa (Figure 4 **E, Figure S6**). Our results showed that a dose of 1 mg/kg of EV‐GLA was able to reduce Gb3 deposits to wild type (WT) levels (*P* = 0.333), hydrolyzing four times more Gb3 than free GLA at the same concentration (*P* = 0.0286). Reduction of Gb3 levels was also observed by immunofluorescence in the kidneys of KO mice treated with EV‐GLA (Figure 4 **F**).

### 2.4 EV‐GLA cross the BBB and reduce Gb3 deposits in brain

**FIGURE 5:**
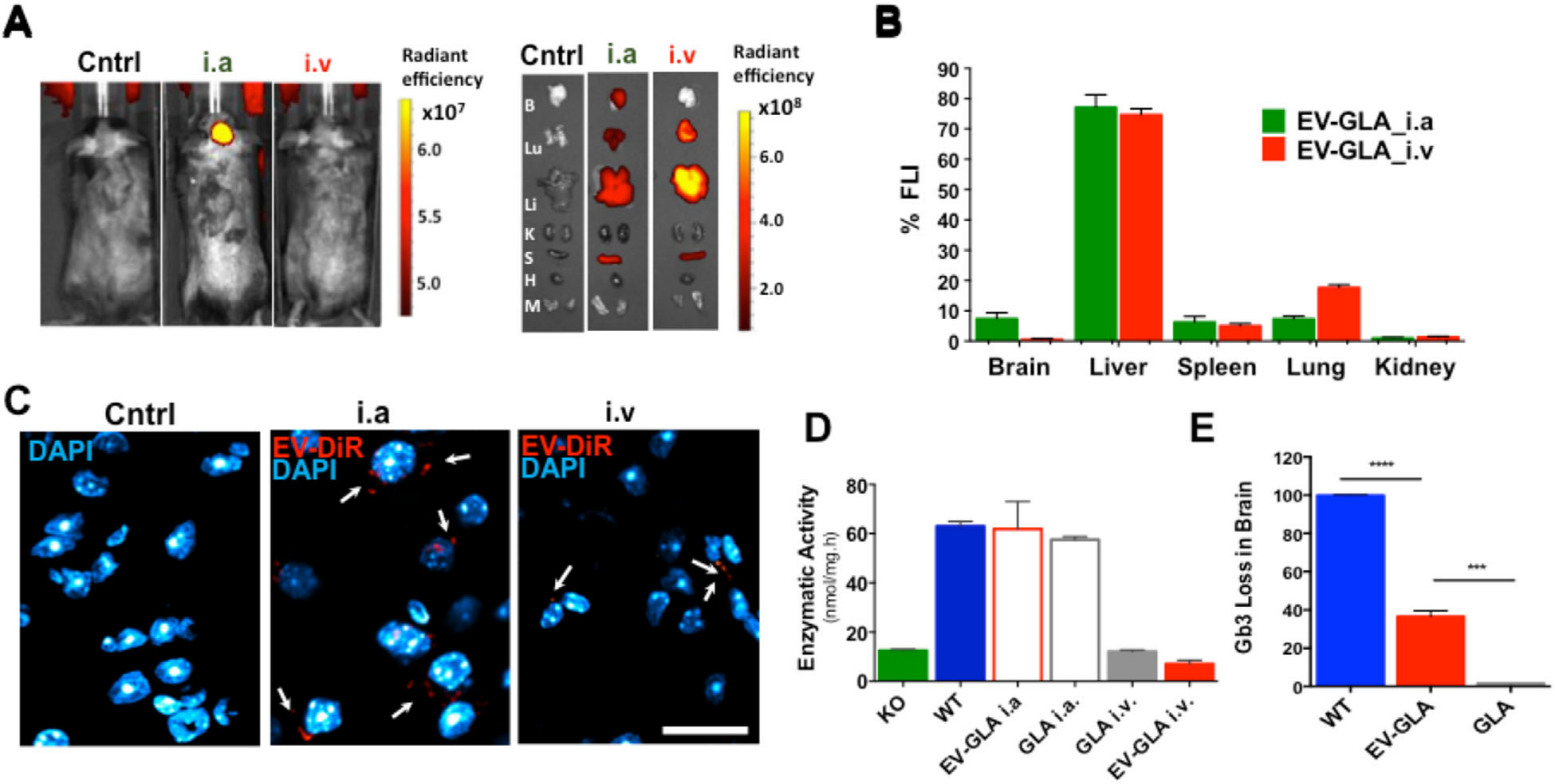
EV‐GLA in the brain parenchyma. **A)** In vivo (left panel) and ex vivo (right panel) fluorescence imaging (FLI) of Fabry KO mice receiving either intra‐arterial (i.a.) or intravenous (i.v.) administration of DiR‐labelled EV‐GLA (1 mg/kg of GLA) compared to the non‐treated controls (Cntrl). Ex vivo imaging included brain (B), lungs (Lu), liver (Li), kidneys (K), spleen (S), heart (H) and muscle (M). **B)** FLI signal quantification comparing biodistribution of EV‐GLA after i.v. or i.a. administration. **C)** Confocal images of brain parenchyma showing DiR fluorescent signal of EV‐GLA (red) and DAPI‐labelled cell nuclei (blue). Magnification bar corresponds to 20 μm. **D)** GLA enzymatic activity 1 h post‐administration in brains of Fabry KO mice treated with GLA or EV‐GLA via i.v. or i.a. administrations **E)** Loss of Gb3 in KO mice treated i.v. with a single dose of EV‐GLA, as measured by LC‐HRMS 1 week after dosing

Crossing the BBB to reach the brain parenchyma is hampered when performing i.v. administration of lysosomal proteins. This prevents the use of current ERT in diseases affecting the central nervous system. However, there are several examples of EVs crossing the BBB in the literature, most of them dealing with the delivery of siRNA or microRNA in EVs after intranasal administration. (Guo et al., 2019; Haney et al., 2015) Among them, those using i.v. administration of EVs took profit of the natural brain tropism of EVs derived from brain endothelial cells (Yang et al., 2015), used macrophage derived EVs in diseases coursing with neuroinflammation (Haney et al., 2019; Yuan et al., 2017) or modified EVs to overexpress BBB crossing peptides (Alvarez‐Erviti et al., 2011; Yang et al., 2017). Therefore, in an attempt to evaluate if EV‐GLA would overcome this barrier, we explored the brain accumulation of fluorescently labelled EVs administered through, i.v. by tail administration and intra‐arterially (i.a.) through the cannulation of the external carotid artery (ECA). While in the i.v. route, exogenously administered compounds only reach the brain circulation after passing through the heart and lungs, in the i.a. administration, cannulation of the ECA allows higher exposure of the compound to brain endothelia (Joshi et al., 2008). In this second biodistribution assay, fluorescent in vivo imaging showed strong DiR fluorescent signal in the brain of mice administered through the carotid (Figure 5 **A, B**), precisely in the hemisphere where arterial cannulation was performed. Ex vivo images were obtained after withdrawing the circulating blood and perfusing the animals with PBS. Our data confirmed that fluorescent signal reached brain parenchyma to a significant extent through the i.a. route (7.49% ± 2.32% of total fluorescence intensity). On the contrary, brain accumulation of DiR after i.v. administration could not be distinguished from background signal. In agreement to this, DiR signal was also located in brain parenchyma of EV‐treated mice, 1 h post‐administration, using confocal microscopy. Fluorescent signal was intense in the case of mice that received the EV‐GLA by arterial infusion, and much lower, although detectable, in animals treated intravenously (Figure 5 **C**).

We wondered whether the i.a. administration could also facilitate the crossing of the BBB. To confirm this hypothesis, GLA KO animals were i.a. or i.v. treated with EV‐GLA and also free recombinant GLA enzyme that is known to be unable to cross the BBB in conventional i.v. ERT. The enzymatic activity of GLA was measured in brain tissues 1 h post‐administration. Accordingly, no GLA enzymatic activity could be detected in brains of KO mice administered i.v. with free GLA or with EV‐GLA. However, significant enzyme activity was observed when the naked enzyme and EV‐GLA were administered i.a. (Figure 5 **D**). These data suggest that GLA (free and EV‐GLA) cross the BBB in higher amount when using intra‐arterial administration instead of i.v. Nonetheless, because the sensitivity of the techniques used to measure GLA activity and biodistribution in the brain have important limitations, we also evaluated Gb3 levels in mice, 1 week after a single i.v. administration of GLA and EV‐GLA (1 mg/kg of GLA, Figure 5 **E** and **Figure S6**). Remarkably, our results clearly showed that EV‐GLA was able to significantly reduce Gb3 deposits in a 36.6 +/‐ 2.97% while free GLA did not induce any reduction of the substrate (*P*  = 0.0009). Overall these results indicate that i.v. administered EV‐GLA is able to reach the brain parenchyma and restore the therapeutic activity at the lysosomal cell compartment. Noteworthy, we show here that EVs, derived from non‐cancerous and non‐dendritic cells and with no specific modifications to reach the brain, are able to cross the BBB after i.v. administrations. The avidity of GLA protein for lipids might explain, at least in part, its ability to trespass the hematoenephalic barrier (Spencer & Verma, 2007) although the exact mechanisms used by EVs to cross the BBB need further investigation.

## 3 CONCLUSION

Protein‐loaded EVs directly obtained from mammalian cell factories and isolated by polymer co‐precipitation methods work as highly efficient protein delivery platforms for ERT in lysosomal disorders. EVs from CHO and HEK cells transfected with GLA and SGSH genes, respectively, are biocompatible, withstand lyophylization and contain high quantities of the recombinant enzymes with increased biological activity. This activity is remarkably higher than the one exhibited by the soluble recombinant proteins in cell supernatants. Further, in Fabry disease mice models, a single dose of GLA containing EVs outperform the activity of the non‐encapsulated enzyme and the clinical marketed reference. After a single intravenous administration, EV‐GLA significantly reduces disease biomarkers Gb3 and LysoGb3 in target tissues including kidneys and brain parenchyma. This proof‐of‐concept work sets the basis for a potential implementation of EVs‐driven replacement therapy in different LSDs, opens the way for a potential treatment strategy in disorders showing CNS involvement.

## 4 EXPERIMENTAL SECTION

*Plasmid preparation and mammalian cell line transfection*: The alpha‐galactosidase A (GLA) gene was obtained from the commercial vector pReceiver‐M10 (EX‐Q0172‐M10, OmicsLink ORF Expression Clone), coding for the cDNA version of the GLA gene (NM_000169), with c‐myc and 6xHis tags fused to the C‐terminus for detection and purification purposes. The gene was subcloned to pOptiVEC‐TOPO (Invitrogen, Themofisher) that allows the expression of the gene of interest along with the dihydrofolate reductase (DHFR) as auxotrophic selection marker. CHO DG44 *dhfr* cells (a dihydrofolate reductase deficient cell line) were transfected (FreeStyle MAX, Thermofisher) and further selected under methotrexate hydrate (MTX, Sigma) up to 4 μM. A single clone (namely CHO‐DG44‐GLA clone #3) was isolated and cryopreserved. For EVs harvesting, CHO‐DG44‐GLA clone #3 was grown in CD OptiCHO medium with 8 mm L‐Glutamine to a final cell concentration of 2 × 10^6^ cells/ml. Cell supernatants containing EVs and soluble, free GLA enzyme were harvested by centrifugation at 3900 rpm for 15 min.

The sequence of human N‐sulphoglucosamine sulphohydrolase (SGSH, BC047318.1) was cloned into the pTriEx1.1‐Hygro vector. Due to the cloning strategy, an extra triplet encoding for an alanine residue was added after the first methionine codon, obtaining a final SGSH protein, with 510 aminoacids in length and a molecular weight of 57.7 kDa. To obtain EVs, SGSH protein was transiently overexpressed in human embryonic kidney cell line (HEK293FT). Briefly, plasmid DNA was polypelexed with polyethylenimine (PEI) at a ratio 1:3 (w/w) in OptiMEM serum free media and added to cells cultured in exosome‐free media (Gibco). Four hours after transfection, valproic acid (4 mm, Sigma‐Aldrich) was added to improve protein expression. Supernatants were collected for EVs isolation after 72 h of incubation at 37°C and 5% CO_2_ in a humidified incubator. Commercially available SGSH protein (Bionova) was purchased to compare the enzymatic activity of the SGSH purified from EVs.

*Purification of EVs*: Supernatants (SN) coming from protein producing cell cultures were used to isolate EVs. In detail, cryovials of CHO‐DG44‐GLA clone #3 (containing 10 million total cells) were thawed and expanded under standard procedures. Briefly, in each passage, cells were diluted to a final cell density of 1 × 10^6^ viable cells/ml. In the last passage before supernatant harvesting, cells were diluted to a final cell concentration of 2 × 10^6^ cells/ml. Finally, supernatant containing extracellular vesicles and soluble, free enzyme was harvested by centrifuging the cell culture at 3900 rpm for 15 min. SN were centrifuged at 300 g at 4°C for 10 min in order to remove dead cells. Additional centrifugation steps at increasing centrifugation speed 2000 g, 10 min 4°C and 10,000 g 20 min 4°C were carried out to remove potential sub‐cellular debris. Clarified SNs were concentrated via VIVAspin 30,000 KDa (Sartorius) centrifugation, 7000 g, and 10 min 4°C. Concentration factors varied from 50X to 100X depending on the sample. EVs were precipitated from concentrated SN through the addition 1:1 (v/v) of ‘Total Exosome Isolation Reagent’, (Invitrogen) and overnight incubation at 4°C. Samples were centrifuged at 16,000 g for 1 h at 4°C and pellets containing the EVs resuspended in PBS. SN of non‐transfected CHO cells were also subjected to the same procedures to obtain control EVs (EV‐Control)

Soluble, free GLA protein with the cmyc‐ and His‐tag of CHO cell supernatants was purified by affinity chromatography with a HisTrap Excel, 5 ml column (GE Healthcare) according to the manufacturer's instructions. Briefly, supernatant was directly injected to the column and later eluted by applying an imidazole gradient (20 to 500 mM). Protein was dialyzed against 0.01 M acetic acid (pH 5.5) and then centrifuged (3900 rpm, 15 min) and filtered (0.2 μm) to discard putative aggregates. Purified enzyme was stored at ‐80°C for further uses.

*EVs characterization*: Morphometric assessment of purified EVs was carried out by CryoTEM analysis. A 3 μl drop of the samples were deposited onto holey carbon on a 400‐mesh copper grid, previously treated by glow discharge. The grid was mounted on a plunger (Leica EM GP), water excess removed by blotting with filter paper and EVs suspension straightforward vitrified by rapid immersion in liquid ethane. Samples were mounted on a Gatan 626 cryo‐transfer system and inserted into a Jeol JEM 2011 Cryo‐electron microscope. It was operated at 200 kV using different degrees of defocus (500–900 nm) to obtain an adequate phase contrast. Images were recorded using a Gatan Ultrascan US1000 CCD camera and further processed using ImageJ NIH software (Eliceiri et al., 2012). EVs diameters were measured and size distributions generated from at least 250 particles from random fields. Particle size distribution and concentration were further studied by Nanoparticle Tracking Analysis (NTA) with Nanosight NS300 (Malvern Panalytical, Malvern, UK). Appropriate dilution of the sample in PBS was performed in order to adjust the range of particles per frame to the working range of the system (10^6^–10^9^ particle/ml). A total of 3 videos were captured at cell temperature of 22°C and syringe speed of 30. >1400 tracks were acquired per video. Data was further processed using NanoSight Software NTA 3.1 Build 3.1.46 with a detection threshold of 5.

EVs or cell lysates were prepared by diluting EVs samples 1:5 in CelLytic reagent (Sigma), homogenized ultrasonic homogenizer (Labsonic M, Sartorious), and total protein amount was determined by BCA method (Pierce, Rockford, IL, USA). GLA and SGSH quantifications were performed by western blot, using known amounts of recombinant proteins, algadidase alfa (Replagal, Shire) or recombinant SGSH (rSGSH, Bionova) and specific antibodies against GLA (ProteinTech) and SGSH (Sigma) at 1:2,000 dilution. Antibodies against CD63 (1:200, SantaCruz), CD81 (1:200, SantaCruz), TSG101 (1:1000, Abcam), β‐tubulin (1:1,000, Invitrogen), cystinosin (1:1000, Mybiosource) and GBA (1:1000, Abcam) were also used to characterize EVs. 20 μg of total protein was loaded per lane with the exception of β‐tubulin and GLA detection in which 10 μg of total protein were employed. Secondary anti‐mouse HRP‐conjugated antibody (1:10,000, Dako) was developed using Immobilon reagent (Millipore), and images were digitally acquired (LI‐COR Odyssey Fc imaging system) and quantified (ImageJ NIH). Protease resistance was determined by incubating 1 μg of GLA and equivalent amounts of its EV‐GLA counterpart in 0.2% trypsin at 37°C for 45 min. After Trypsin digestion appropriate volumes were loaded into 10% PAA gels and GLA immunodetected as detailed above. Densitometry analysis of GLA bands was carried out using ImageJ NIH software. Band signal was defined with the IntDen or integrated density, an Image J descriptor consisting in the product of mean intensity and band area.

*Enzymatic activity determinations*: GLA enzymatic activity was determined fluorometrically as described previously ^16,17^ by monitoring the transformation of the substrate 4‐Methylumbelliferyl β‐D‐galactopyranoside (4‐MUG, Sigma) into fluorescent 4‐methylumbelliferone (4‐MU). SGSH enzymatic activity was assessed by two consecutive enzymatic reactions as previously detailed by Karpova and co‐workers, (Karpova et al., 1996) using the same standardized procedure followed in clinical diagnosis of MPSIIIA. Accordingly, substrate 4‐methylumbelliferyl‐α‐d‐N‐sulfoglucosaminide (4MU‐αGlcNS, Enantia) was incubated with EV‐SGSH and rSGSH samples for 17 h at 37°C to generate 4‐MU‐αGlcNH2. Fluorescent 4‐MU was then released by α‐glycosidase (Sigma) after 24 h of incubation at 37°C. Different dilutions of the EV and protein samples were tested to ensure that obtained 4‐MU fall within the linear range of the technique. Final values for enzymatic activity were given in μmol of 4‐MU product generated per hour and mg of GLA or SGSH enzyme or referred to total protein content when dealing with tissue activities.

*Cell culture conditions*: HEK293T cells (CRL‐3216, ATCC) were routinely maintained in RPMI medium supplemented with 10% foetal bovine serum (Life Technologies, Paisley, UK), 2 mm L‐Glutamine (Life Technologies), 1 X Non‐essential aminoacids (Life Technologies), 1X antimycotic‐antibiotic (Life Technologies) solution at 37°C and 5% CO_2_. Mouse endothelial aortic cells (MAEC) were isolated from descending aorta of GLA deficient mice (GlatmKul1) at the ICTS NANBIOSIS (U20) following procedures previously established procedures (Giannotti et al., 2016; Nazarenus et al., 2015; Shu et al., 2005). Endothelial origin of isolated cells was confirmed by CD105 staining (12‐1051, eBioscience) and flow cytometry (FacScalibur, Becton Dickinson).

*EV fluorescent labelling*: DiOC, DiD, or DiR (Invitrogen) were incubated to a concentration of 250 μg/ml with EVs for 30 min at 37°C. Samples were dialyzed (Slide‐A‐Lyzer MINI Dialysis Device, 3.5K MWCO, 0.1 ml, Thermo Scientific) against PBS, to remove unbound fluorophore and avoid potential artifacts. The absence of significant fluorescent signal after dialysis on labelling controls, namely diluent at an equivalent fluorophore concentration than the used to label EVs, was confirmed (data not shown).

*Flow cytometry*: DiOC labelled EVs were added to HEK293 cultures, 2.5 μg EVs/ml, and incubated for 15, 30, 60 and 240 min. After incubation plates were treated with trypsin 0.05 % (w/v) (Biological Industries) to detach cells, and neutralized in complete RPMI medium supplemented with DAPI (1 μg/ml final concentration), in order to remove dead cells from analysis. Cell fluorescence intensity was analyzed in a LSR Fortessa flow cytometer (Beckton Dickinson). Data were further processed using FCS express 4 software (De novo software) and median fluorescence intensity represented. All time points were tested in duplicate.

*Confocal microscopy*: HEK293 or MAEC cells were seeded in an 8 chambered coverglass (Lab‐Tek II, Eppendorf) and incubated 24 h at 37°C and 5% CO_2_. Then, 2.5 μg/ml DiOC labelled EVs were added and incubated for 4 h. Cell medium was removed and cells were stained with 5 μg/ml Cell Mask™ (Invitrogen) and fixed in 4% paraformaldehyde (PFA). Image acquisition was carried out by spectral confocal microscopy FV1000 (Olympus) with a PLAPON 60XO objective. Z projections sections were acquired with a step size between 0.4 and 0.6 μm and further reconstructed using ImageJ.

*STORM*: HEK293 cells were incubated with 2.5 μg/ml DiD‐labelled EVs during 4 h, 8 h or O.N in cell culture conditions. After fixation with 4% PFA, cells were permeabilized (300 mm sucrose, 50 mm NaCl, 20 mm HEPES, 3 mm MgCl_2_ and 0.5% Triton X100, 5 min at 4°C) and blocked with 1% BSA in PBS, prior to the incubation with anti‐LAMP1 (1:1000, Abcam), 1 h at RT. After washing, Alexa 488‐labeled anti‐mouse secondary antibody (1:200, Invitrogen) was incubated for an additional hour. Samples were imaged in STORM buffer (5% w/v glucose, 100 mm cysteamine, 0.5 mg/ml glucose oxidase and 40 μg/ml catalase in PBS) to ensure an adequate photoswitching of the fluorophores. DiD‐labelled EVs were imaged with a 647 nm laser (160 mW) and Alexa 488 immunolabelled lysosomes with a 488 nm laser (80 mW) using NIS‐Elements software in Nikon Eclipse Ti microscope (Nikon Europe, Amsterdam). The sample was illuminated using a total internal reflection fluorescence (TIRF) alignment system and the z‐level was kept constant by Nikon perfect focus system. Fluorescence was collected by a Nikon 100x, 1.49 NA oil immersion objective and images acquired with a Hamamatsu 19 ORCA‐ Flash 4.0 camera. For each channel 20,000 frames were acquired and analyzed by fitting a 2D Gaussian function to obtain the localizations of fluorophores. Co‐localization was determined using the *Analyze Particles* tool in ImageJ setting a minimum size of 0.02 μm^2^ (corresponding to a circular object with an 80 nm radius) and no constrains on circularity. The centre positions of lysosome channel images and EVs channel images were loaded onto a custom Matlab script considering a particular EV being inside a lysosome when its centre was closer than 50 nm to the centre of a lysosome.

*In vitro efficacy (NBD‐Gb3 assays)*: MAEC at passages 2 to 5 were seeded in 24 well plates 24 h after seeding 8 μM of fluorescent N‐Dodecanoyl‐NBD‐ceramide trihexoside (NBD‐Gb3, Matreya LCC) was added to the cultures along with specified concentrations of tested compounds. After 48 h incubation, cells were trypsinized and NBD‐Gb3 fluorescent signal was analyzed by flow cytometry (FacsCalibur, Beckton Dickinson) and FCS Express v4 software. To calculate the percentage of NBD‐Gb3 signal, fluorescent signal in control cells (without treatment) was established as 100% and the values accordingly normalized.

*Lyophilization*: Aliquots of 150 μL of EV‐GLA (8.4 mg /ml of total protein), previously frozen at ‐80°C, were lyophilized in a VirTis sentry 2.0 freeze dryer (Sp scientific). No additives or cryopreservants were added. Morphology and functionality of EVs post‐lyophilization was assessed as described above.

*In vivo assays*: All animal experimentation, including the obtention of MAEC, was performed following procedures previously approved by the Ethical Committee for the Use of Experimental Animals (CEEA) at the Vall d'Hebron Research Institute (VHIR), Barcelona and the local government (CEA‐OH/9572). Male GLA deficient mice (GlatmKul1, C57BL/6 background) with ages ranging from 2 to 4 months were used for biodistribution and efficacy studies.

*Biodistribution*: In the first assay, each mouse (*n *= 3) was treated by tail vein injection with 100 μg, total protein (5 μg of GLA/mouse), of DiR‐labelled EVs and euthanized 1 h post‐administration. In the second assay, i.v. (tail vein) and i.a administrations were compared in mice (n = 3 per group) treated with 1 mg/kg in GLA equivalents of DiR‐labelled EVs (c.a. 35 μg of GLA/mouse) and euthanized 1 h post‐administration. I.a. administrations were adapted for mice following previously described procedures in rats and using procedures (Janowski et al., 2013). In both biodistribution assays, fluorescent images of ex vivo tissues were acquired (IVIS Spectrum), quantified (Living Image software) and referred to the weight of the wet tissue when required. EV visualization was performed in PFA fixed and OCT embedded tissues sections counterstained with DAPI (Sigma‐Aldrich) by confocal microscopy. GLA enzymatic activity was measured in harvested tissues, following procedures described above, a referred to the total protein content determined by the BCA method (Pierce, ThermoFisher).

*Immunohistochemistry*: The presence of GLA antigen in liver was analyzed by pre‐treating paraffin‐embedded formalin fixed sections with 100 mm citrate buffer (pH 9) in a pressure cooker. Sections were incubated with 10% normal goat serum (NGS) in antibody diluent (1% BSA in 100 mm Tris buffer) and then of 1:150 dilution of anti‐GLA antibody (HPA00237, Sigma). Secondary antibody consisted in a HRP conjugated system (EnVision+ System‐HRP Labelled Polymer anti‐Mouse), which was later visualized with DAB and counterstained with Harris haematoxylin. Gb3 deposits were identified using anti‐Gb3 monoclonal antibody (1:50, Clone BGR23, Amsbio) in paraformaldehyde fixed and OCT embedded frozen tissues. Sections were incubated with a secondary antibody (1:1000, A10684, Invitrogen) and counterstained with DAPI and rodhamine‐phalloidin (Sigma‐Aldrich). Visualization was performed by confocal microscopy as explained above.

*Efficacy assays in vivo*: Fabry KO mice (*n *= 24) were randomized in four different groups (vehicle, agalsidase‐alfa, GLA and EVs‐GLA), and received a single doses of 1 mg/kg of GLA protein. Vehicle treated animals receive the corresponding volume of PBS and additional group of non‐treated WT male littermates was also included. Animals were euthanized 24 h or 1 week after dosing. Tissue samples were snap frozen and kept at ‐80°C upon analysis. Gb3 and LysoGb3 levels were determined with LC‐HRMS at Institute of Advanced Chemistry of Catalonia (IQAC‐CSIC) (Supplementary Information).

*Statistical analysis*: All graphs show the mean and the SEM, unless otherwise stated. For statistical analysis, whenever data followed a normal distribution (Kolmogorov‐Smirnov test) unpaired Student's *t*‐test was used for peer comparisons between EV‐GLA and controls. Alternatively, non‐parametric Mann‐Whitney test was applied. The significance threshold was established at *P*  < 0.05, and significance levels were schematically assigned *(0.01 ≤ *P*  < 0.05), **(0.001 ≤ *P*  < 0.01), ***(0.0001 ≤ *P*). All the analyses and graphs were performed using GraphPad Prism 6 software (GraphPad, San Diego).

## 5 ASSOCIATED CONTENT

**Supporting Information**. Methodology for EV isolation methods by TFF, sucrose cushion and iodixanol gradient, lyophilization and LC‐HRMS determinations, as well as results on EV isolation, characterization, cell internalization and in vivo safety and efficacy are available as supplementary information.

## CONFLICTS OF INTEREST

Joaquin Seras‐Franzoso, José Luis Corchero, Simó Schwartz Jr and Ibane Abasolo are co‐applicants of a patent describing the use of engineered EV for the producing highly active enzymes (P201930056, 24/01/2019).

## AUTHOR CONTRIBUTIONS

The manuscript was written through contributions of all authors. All authors have given approval to the final version of the manuscript.

## FUNDING SOURCES

This study has been supported by ISCIII (PI18_00871 co‐founded by Fondo Europeo de Desarrollo Regional (FEDER)), and CIBER‐BBN (EXPLORE) granted to IA. Different CIBER‐BBN units of ICTS ‘NANBIOSIS’ have participated in this work (https://www.nanbiosis.es/platform-units/), more specifically the U1/Protein Production Platform for protein purification, Unit 6 for NTA analysis and TFF purification and U20/FVPR for in vivo assays.

## Supporting information

Supporting information.

## References

[jev212058-bib-0001] Alvarez‐Erviti, L. , Seow, Y. , Yin, H. , Betts, C. , Lakhal, S. , & Wood, M. J. A. (2011). Delivery of SiRNA to the mouse brain by systemic injection of targeted exosomes. Nature Biotechnology, 29(4), 341–345. 10.1038/nbt.1807 21423189

[jev212058-bib-0002] Bandaranayake, A. D. , & Almo, S. C. (2014). Recent advances in mammalian protein production. FEBS Letters, 588(2), 253. 24316512 10.1016/j.febslet.2013.11.035 PMC3924552

[jev212058-bib-0003] Batrakova, E. V. , & Kim, M. S. (2015). Using exosomes, naturally‐equipped nanocarriers, for drug delivery. Journal of Controlled Release, 219, 396–405. 26241750 10.1016/j.jconrel.2015.07.030 PMC4656109

[jev212058-bib-0004] Beck, M. , Ricci, R. , Widmer, U. , Dehout, F. , De Lorenzo, A. G. , Kampmann, C. , Linhart, A. , Sunder‐Plassmann, G. , Houge, G. , Ramaswami, U. , Gal, A. , & Mehta, A. (2004). Fabry disease: overall effects of agalsidase alfa treatment. European Journal of Clinical Investigation, 34(12), 838–844. 15606727 10.1111/j.1365-2362.2004.01424.x

[jev212058-bib-0005] Bobrie, A. , Colombo, M. , Krumeich, S. , Raposo, G. , & Théry, C. (2012). Diverse subpopulations of vesicles secreted by different intracellular mechanisms are present in exosome preparations obtained by differential ultracentrifugation. Journal of Extracellular Vesicles, 1, 18397. 10.3402/jev.v1i0.18397 PMC3760636 24009879

[jev212058-bib-0006] Bodary, P. F. , Shayman, J. a , & Eitzman, D. T. (2007). Alpha‐Galactosidase A in vascular disease. Trends in Cardiovascular Medicine, 17(4), 129–133. 17482095 10.1016/j.tcm.2007.02.006

[jev212058-bib-0007] Bosch, S. , de Beaurepaire, L. , Allard, M. , Mosser, M. , Heichette, C. , Chrétien, D. , Jegou, D. , & Bach, J. ‐ M. (2016). Trehalose prevents aggregation of exosomes and cryodamage. Scientific Reports, 6(1), 36162. 27824088 10.1038/srep36162 PMC5099918

[jev212058-bib-0008] Busatto, S. , Vilanilam, G. , Ticer, T. , Lin, W. ‐ L. , Dickson, D. , Shapiro, S. , Bergese, P. , & Wolfram, J. (2018). Tangential flow filtration for highly efficient concentration of extracellular vesicles from large volumes of fluid. Cells, 7(12), 273. 10.3390/cells7120273 PMC6315734 30558352

[jev212058-bib-0009] Cabrera, I. , Abasolo, I. , Corchero, J. L. , Elizondo, E. , Gil, P. R. , Moreno, E. , Faraudo, J. , Sala, S. , Bueno, D. , González‐Mira, E. , Rivas, M. , Melgarejo, M. , Pulido, D. , Albericio, F. , Royo, M. , Villaverde, A. , García‐Parajo, M. F. , Schwartz, S. , Ventosa, N. , & Veciana, J. (2016). α‐Galactosidase‐A loaded‐nanoliposomes with enhanced enzymatic activity and intracellular penetration. Advanced Healthcare Materials, 5(7), 829–840. 26890358 10.1002/adhm.201500746

[jev212058-bib-0010] Concolino, D. , Deodato, F. , & Parini, R. (2018). Enzyme replacement therapy: efficacy and limitations. Italian Journal of Pediatrics, 44, 120. 30442189 10.1186/s13052-018-0562-1 PMC6238252

[jev212058-bib-0011] Corso, G. , Mäger, I. , Lee, Y. , Görgens, A. , Bultema, J. , Giebel, B. , Wood, M. J. A. , Nordin, J. Z. , & Andaloussi, S. El (2017). Reproducible and scalable purification of extracellular vesicles using combined bind‐elute and size exclusion chromatography. Scientific Reports, 7 10.1038/s41598-017-10646-x PMC5599601 28912498

[jev212058-bib-0012] Desnick, R. J. , Ioannou, Y. A. , & Eng, C. M. (2001). Alpha‐Galactosidasa A Deficiency: Fabry Disease. In The Metabolic and Molecular Bases of Inherited Disease, C. R., Scriver , A. L., Beaudet , W. S., Sly , D., Valle , B., Childs , K. W., Kinzler , & B., Vogelstein Eds., New York: McGraw‐Hill, pp 3733–3774.

[jev212058-bib-0013] Do, M. A. , Levy, D. , Brown, A. , Marriott, G. , & Lu, B. (2019). Targeted delivery of lysosomal enzymes to the endocytic compartment in human cells using engineered extracellular vesicles. Scientific Reports, 9(1), 9. 31754156 10.1038/s41598-019-53844-5 PMC6872767

[jev212058-bib-0014] Eliceiri, K. , Schneider, C. A. , Rasband, W. S. , & Eliceiri, K. W. (2012). NIH image to ImageJ : 25 years of image analysis. Nature Methods, 9(7), 671–675. 22930834 10.1038/nmeth.2089 PMC5554542

[jev212058-bib-0015] Eng, C. M. , Guffon, N. , Wilcox, W. R. , Germain, D. P. , Lee, P. , Waldek, S. , Caplan, L. , Linthorst, G. E. , & Desnick, R. J. (2001). International Collaborative Fabry Disease Study Group. Safety and efficacy of recombinant human alpha‐Galactosidase A replacement therapy in Fabry's disease. New England Journal of Medicine, 345(1), 9–16. 10.1056/NEJM200107053450102 11439963

[jev212058-bib-0016] Fedele, A. O. , Isenmann, S. , Kamei, M. , Snel, M. F. , Trim, P. J. , Proud, C. G. , & Hopwood, J. J. (2018). Lysosomal N‐Acetyltransferase interacts with ALIX and is detected in extracellular vesicles. Biochimica et Biophysica Acta (BBA) ‐ Molecular Cell Research, 1865(10), 1451–1464. 29981367 10.1016/j.bbamcr.2018.07.001

[jev212058-bib-0017] Fleury, A. , Martinez, M. C. , & Le Lay, S. (2014). Extracellular vesicles as therapeutic tools in cardiovascular diseases. Frontiers in Immunology, 5, 370. 10.3389/fimmu.2014.00370 25136343 PMC4120684

[jev212058-bib-0018] Frank, J. , Richter, M. , de Rossi, C. , Lehr, C. M. , Fuhrmann, K. , & Fuhrmann, G. (2018). Extracellular vesicles protect glucuronidase model enzymes during freeze‐drying. Scientific Reports, 8, 12377. 10.1038/s41598-018-30786-y 30120298 PMC6098026

[jev212058-bib-0019] Gaffke, L. , Pierzynowska, K. , Piotrowska, E. , & Węgrzyn, G. (2018). How close are we to therapies for Sanfilippo disease? Metabolic Brain Disease, 33, 1–10, Springer New York LLC 28921412 10.1007/s11011-017-0111-4 PMC5769821

[jev212058-bib-0020] Geisse, S. , & Fux, C. (2009). Chapter 15 recombinant protein production by transient gene transfer into mammalian cells. Methods in Enzymology, 463, 223–238. 19892175 10.1016/S0076-6879(09)63015-9

[jev212058-bib-0021] Giannotti, M. I. , Abasolo, I. , Oliva, M. , Andrade, F. , García‐Aranda, N. , Melgarejo, M. , Pulido, D. , Corchero, J. L. , Fernández, Y. , Villaverde, A. , Royo, M. , García‐Parajo, M. F. , Sanz, F. , & Schwartz, S. (2016). Highly versatile polyelectrolyte complexes for improving the enzyme replacement therapy of lysosomal storage disorders. ACS Applied Materials & Interfaces, 8(39), 25741–25752. 27610822 10.1021/acsami.6b08356

[jev212058-bib-0022] Goetzl, E. J. , Boxer, A. , Schwartz, J. B. , Abner, E. L. , Petersen, R. C. , Miller, B. L. , Kapogiannis, D. (2015). Altered lysosomal proteins in neural‐derived plasma exosomes in preclinical Alzheimer disease. Neurology 85(1), 40–47. 26062630 10.1212/WNL.0000000000001702 PMC4501943

[jev212058-bib-0023] Guérard, N. , Oder, D. , Nordbeck, P. , Zwingelstein, C. , Morand, O. , Welford, R. W. D. , Dingemanse, J. , & Wanner, C. L. (2018). An iminosugar for substrate reduction therapy: tolerability, pharmacodynamics, and pharmacokinetics in patients with Fabry disease on enzyme replacement. Clinical Pharmacology and Therapeutics, 103(4), 703–711. 28699267 10.1002/cpt.790

[jev212058-bib-0024] Guo, S. , Perets, N. , Betzer, O. , Ben‐Shaul, S. , Sheinin, A. , Michaelevski, I. , Popovtzer, R. , Offen, D. , & Levenberg, S. (2019). Intranasal delivery of mesenchymal stem cell derived exosomes loaded with phosphatase and tensin homolog SiRNA repairs complete spinal cord injury. ACS Nano 13, 10015–10028. 31454225 10.1021/acsnano.9b01892

[jev212058-bib-0025] Haney, M. J. , Klyachko, N. L. , Zhao, Y. , Gupta, R. , Plotnikova, E. G. , He, Z. , Patel, T. , Piroyan, A. , Sokolsky, M. , Kabanov, A. V. , & Batrakova, E. V. (2015). Exosomes as drug delivery vehicles for Parkinson's disease therapy. Journal of Controlled Release 207, 18–30 25836593 10.1016/j.jconrel.2015.03.033 PMC4430381

[jev212058-bib-0026] Haney, M. J. , Klyachko, N. L. , Harrison, E. B. , Zhao, Y. , Kabanov, A. V. , Batrakova, E. V. (2019). TPP1 delivery to lysosomes with extracellular vesicles and their enhanced brain distribution in the animal model of batten disease. Advanced Healthcare Materials, 8(11), 1801271. 10.1002/adhm.201801271 PMC6584948 30997751

[jev212058-bib-0027] Hansen, M. S. , Gadegaard, I. S. E. , Arnspang, E. C. , Blans, K. , Nejsum, L. N. , & Rasmussen, J. T. (2020). Specific and non‐invasive fluorescent labelling of extracellular vesicles for evaluation of intracellular processing by intestinal epithelial cells. Biomedicines, 8(7), 211. 10.3390/biomedicines8070211 PMC7400383 32674302

[jev212058-bib-0028] Hsu, J. , Bhowmick, T. , Burks, S. R. , Kao, J. P. Y. , & Muro, S. (2014). Enhancing biodistribution of therapeutic enzymes in vivo by modulating surface coating and concentration of ICAM‐1‐targeted nanocarriers. Journal of Biomedical Nanotechnology, 10 (2), 345–354. 24738342 10.1166/jbn.2014.1718 PMC4000549

[jev212058-bib-0029] Huang, J. , Khan, A. , Au, B. C. , Barber, D. L. , López‐Vásquez, L. , Prokopishyn, N. L. , Boutin, M. , Rothe, M. , Rip, J. W. , Abaoui, M. , Nagree, M. S. , Dworski, S. , Schambach, A. , Keating, A. , West, M. L. , Klassen, J. , Turner, P. V. , Sirrs, S. , Rupar, C. A., … Medin, J. A. (2017). Lentivector iterations and pre‐clinical scale‐up/toxicity testing: targeting mobilized CD34+ cells for correction of Fabry disease. Molecular Therapy, Methods & Clinical Development, 5, 241–258. 28603745 10.1016/j.omtm.2017.05.003 PMC5453867

[jev212058-bib-0030] Hughes, D. A. , Nicholls, K. , Shankar, S. P. , Sunder‐Plassmann, G. , Koeller, D. , Nedd, K. , Vockley, G. , Hamazaki, T. , Lachmann, R. , Ohashi, T. , Olivotto, I. , Sakai, N. , Deegan, P. , Dimmock, D. , Eyskens, F. , Germain, D. P. , Goker‐Alpan, O. , Hachulla, E. , Jovanovic, A., … Feldt‐Rasmussen, U. (2017). Oral pharmacological chaperone migalastat compared with enzyme replacement therapy in Fabry disease: 18‐month results from the randomised phase III ATTRACT Study. Journal of Medical Genetics, 54(4), 288–296. 27834756 10.1136/jmedgenet-2016-104178 PMC5502308

[jev212058-bib-0031] Janowski, M. , Lyczek, A. , Engels, C. , Xu, J. , Lukomska, B. , Bulte, J. W. M. , & Walczak, P. (2013). Cell size and velocity of injection are major determinants of the safety of intracarotid stem cell transplantation. Journal of Cerebral Blood Flow and Metabolism, 33(6), 921–927. 23486296 10.1038/jcbfm.2013.32 PMC3677113

[jev212058-bib-0032] Joshi, B. S. , de Beer, M. A. , Giepmans, B. N. G. , & Zuhorn, I. S. (2020). Endocytosis of extracellular vesicles and release of their cargo from endosomes. ACS Nano, 14(4), 4444–4455. 32282185 10.1021/acsnano.9b10033 PMC7199215

[jev212058-bib-0033] Joshi, S. , Meyers, P. M. , & Ornstein, E. (2008). Intracarotid delivery of drugs: the potential and the pitfalls. Anesthesiology, 109, Lippincott Williams and Wilkins, pp 543–564. 10.1097/ALN.0b013e318182c81b 18719453 PMC3999414

[jev212058-bib-0034] Karimi, N. , Cvjetkovic, A. , Jang, S. C. , Crescitelli, R. , Hosseinpour Feizi, M. A. , Nieuwland, R. , Lötvall, J. , & Lässer, C. (2018). Detailed analysis of the plasma extracellular vesicle proteome after separation from lipoproteins. Cellular and Molecular Life Sciences 75, 2873–2886 29441425 10.1007/s00018-018-2773-4 PMC6021463

[jev212058-bib-0035] Karpova, E. A. , Voznyi YaV, Keulemans, J. L. , Hoogeveen, A. T. , Winchester, B. , Tsvetkova, I. , & V, van Diggelen, O. P. (1996). A fluorimetric enzyme assay for the diagnosis of Sanfilippo disease type A (MPS IIIA). Journal of Inherited Metabolic Disease, 19(3), 278–285. 8803769 10.1007/BF01799255

[jev212058-bib-0036] Kowal, J. , Arras, G. , Colombo, M. , Jouve, M. , Morath, J. P. , Primdal‐Bengtson, B. , Dingli, F. , Loew, D. , Tkach, M. , & Théry, C. (2016). Proteomic comparison defines novel markers to characterize heterogeneous populations of extracellular vesicle subtypes. Proceedings of the National Academy of Sciences of the United States of America, 113(8), E968–E977. 26858453 10.1073/pnas.1521230113 PMC4776515

[jev212058-bib-0037] Leader, B. , Baca, Q. J. , & Golan, D. E. (2008). Protein therapeutics: A summary and pharmacological classification. Nature Reviews Drug Discovery, 7, 21–39. 18097458 10.1038/nrd2399

[jev212058-bib-0038] Lee, H. J. , Park, H. H. , Sohn, Y. , Ryu, J. , Park, J. H. , Rhee, W. J. , & Park, T. H. (2016). α‐Galactosidase delivery using 30Kc19‐human serum albumin nanoparticles for effective treatment of Fabry disease. Applied Microbiology and Biotechnology, 100(24), 10395–10402. 27353764 10.1007/s00253-016-7689-z

[jev212058-bib-0039] Li, D. , Liu, J. , Guo, B. , Liang, C. , Dang, L. , Lu, C. , He, X. , Cheung, H. Y.‐.S. , Xu, L. , Lu, C. , He, B. , Liu, B. , Shaikh, A. B. , Li, F. , Wang, L. , Yang, Z. , Au, D. W.‐.T. , Peng, S. , Zhang, Z., … Zhang, Ge (2016). Osteoclast‐derived exosomal MiR‐214‐3p inhibits osteoblastic bone formation. Nature Communications, 7, 10872. 10.1038/ncomms10872 PMC4786676 26947250

[jev212058-bib-0040] Li, H. , Pinilla‐Macua, I. , Ouyang, Y. , Sadovsky, E. , Kajiwara, K. , Sorkin, A. , & Sadovsky, Y. (2020). Internalization of trophoblastic small extracellular vesicles and detection of their MiRNA Cargo in P‐Bodies. Journal of Extracellular Vesicles, 9(1), 1812261. 32944196 10.1080/20013078.2020.1812261 PMC7480505

[jev212058-bib-0041] Lobb, R. J. , Becker, M. , Wen, S. W. , Wong, C. S. F. , Wiegmans, A. P. , Leimgruber, A. , & Möller, A. (2015). Optimized exosome isolation protocol for cell culture supernatant and human plasma. Journal of Extracellular Vesicles, 4(1), 27031. 26194179 10.3402/jev.v4.27031 PMC4507751

[jev212058-bib-0042] Medin, J. A. , Khan, A. , Huang, Ju , Barber, D. , Anthony Rupar, C. , Auray‐Blais, C. , Fraser, G. , Fowler, D. H. , Keating, A. , West, M. L. , & Foley, R. (2019). FACTs Fabry gene therapy clinical trial: two‐year data. Molecular Genetics and Metabolism, 126(2), S99.

[jev212058-bib-0043] Mehta, . , Beck, M. , Elliott, P. , Giugliani, R. , Linhart, A. , Sunder‐Plassmann, G. , Schiffmann, R. , Barbey, F. , Ries, M. , Clarke, J. Enzyme replacement therapy with agalsidase alfa in patients with Fabry's disease: an analysis of registry data. Lancet (London, England) 2009, 374(9706), 1986–1996. 10.1016/S0140-6736(09)61493-8 19959221

[jev212058-bib-0044] Meikle, P. J. , Hopwood, J. J. , Clague, A. E. , & Carey, W. F. (1999). Prevalence of lysosomal storage disorders. JAMA, 281(3), 249–254. 9918480 10.1001/jama.281.3.249

[jev212058-bib-0045] Mulcahy, L. A. , Pink, R. C. , & Carter, D. R. F. (2014). Routes and mechanisms of extracellular vesicle uptake. Journal of Extracellular Vesicles, 3, 24641, Co‐Action Publishing. 10.3402/jev.v3.24641 PMC4122821 25143819

[jev212058-bib-0046] Muro, S. (2010). New biotechnological and nanomedicine strategies for treatment of lysosomal storage disorders. Wiley Interdisciplinary Reviews Nanomedicine and Nanobiotechnology, 2(2), 189–204. 20112244 10.1002/wnan.73 PMC4002210

[jev212058-bib-0047] Nazarenus, M. , Abasolo, I. , García‐Aranda, N. , Voccoli, V. , Rejman, J. , Cecchini, M. , Schwartz, S. , Rivera‐Gil, P. , & Parak, W. J. (2015). Polymer capsules as a theranostic tool for a universal in vitro screening assay ‐ The case of lysosomal storage diseases. Particle & Particle Systems Characterization : Measurement and Description of Particle Properties and Behavior in Powders and Other Disperse Systems, 32(11), 991–998. 10.1002/ppsc.201500156

[jev212058-bib-0048] Nizamudeen, Z. , Markus, R. , Lodge, R. , Parmenter, C. , Platt, M. , Chakrabarti, L. , & Sottile, V. (2018). Rapid and accurate analysis of stem cell‐derived extracellular vesicles with super resolution microscopy and live imaging. Biochimica et Biophysica Acta (BBA) ‐ Molecular Cell Research, 1865(12), 1891–1900. 30290236 10.1016/j.bbamcr.2018.09.008 PMC6203808

[jev212058-bib-0049] Platt, F. M. , D'Azzo, A. , Davidson, B. L. , Neufeld, E. F. , & Tifft, C. J. (2018). Lysosomal storage diseases. Nature Reviews Disease Primers, 4(1), 27. 10.1038/s41572-018-0025-4 30275469

[jev212058-bib-0050] Raposo, G. , & Stoorvogel, W. (2013). Extracellular vesicles: exosomes, microvesicles, and friends. Journal of Cell Biology, 200(4), 373–383. 10.1083/jcb.201211138 PMC3575529 23420871

[jev212058-bib-0051] Rombach, S. M. , Twickler, T. B. , Aerts, J. M. F. G. , Linthorst, G. E. , Wijburg, F. A. , & Hollak, C. E. M. (2010). Vasculopathy in patients with Fabry disease: current controversies and research directions. Molecular Genetics and Metabolism, 99(2), 99–108. 19900828 10.1016/j.ymgme.2009.10.004

[jev212058-bib-0052] Safary, A. , Akbarzadeh Khiavi, M. , Mousavi, R. , Barar, J. , & Rafi, M. A. (2018). Enzyme replacement therapies: what is the best option? Bioimpacts, 8(3), 153–157. 30211074 10.15171/bi.2018.17 PMC6128977

[jev212058-bib-0053] Shu, L. , Murphy, H. S. , Cooling, L. , & Shayman, J. a. (2005). An in vitro model of Fabry disease. Journal of the American Society of Nephrology, 16, 2636–2645. 16033856 10.1681/ASN.2005040383

[jev212058-bib-0054] Sly, W. S. , Vogler, C. , Grubb, J. H. , Levy, B. , Galvin, N. , Tan, Y. , Nishioka, T. , & Tomatsu, S. (2006). Enzyme therapy in mannose receptor‐null mucopolysaccharidosis VII mice defines roles for the mannose 6‐phosphate and mannose receptors. Proceedings of the National Academy of Sciences, 103(41), 15172–15177. 10.1073/pnas.0607053103 PMC1622795 17015822

[jev212058-bib-0055] Solomon, M. , & Muro, S. (2017). Lysosomal enzyme replacement therapies: historical development, clinical outcomes, and future perspectives. Advanced Drug Delivery Reviews, 118, 109–134. 28502768 10.1016/j.addr.2017.05.004 PMC5828774

[jev212058-bib-0056] Somiya, M. , Yoshioka, Y. , & Ochiya, T. (2018). Biocompatibility of highly purified bovine milk‐derived extracellular vesicles. Journal of Extracellular Vesicles, 7(1), 1440132. 29511463 10.1080/20013078.2018.1440132 PMC5827637

[jev212058-bib-0057] Spencer, B. J. , & Verma, I. M. (2007). Targeted delivery of proteins across the blood‐brain barrier. Proceedings of the National Academy of Sciences of the United States of America, 104(18), 7594–7599. 17463083 10.1073/pnas.0702170104 PMC1857226

[jev212058-bib-0058] Srivastava, A. , Babu, A. , Filant, J. , Moxley, K. M. , Ruskin, R. , Dhanasekaran, D. , Sood, A. K. , McMeekin, S. , & Ramesh, R. (2016). Exploitation of exosomes as nanocarriers for gene‐, chemo‐, and immune‐therapy of cancer. Journal of Biomedical Nanotechnology, 12(6), 1159–1173. 27319211 10.1166/jbn.2016.2205

[jev212058-bib-0059] Sunder‐Plassmann, G. (2006). Renal Manifestations of Fabry Disease. In Fabry Disease: Perspectives from 5 Years of FOS, A., Mehta , M., Beck , & G., Sunder‐Plassmann Eds., Oxford: Oxford PharmaGenesis. 21290683

[jev212058-bib-0060] Sutaria, D. S. , Badawi, M. , Phelps, M. A. , & Schmittgen, T. D. (2017). Achieving the promise of therapeutic extracellular vesicles: the devil is in details of therapeutic loading. Pharmaceutical Research, 34(5), 1053–1066. 28315083 10.1007/s11095-017-2123-5 PMC5565485

[jev212058-bib-0061] Théry, C. , Amigorena, S. , Raposo, G. , & Clayton, A. (2006). Isolation and characterization of exosomes from cell culture supernatants and biological fluids. Current Protocols in Cell Biology, 30(1), 3.22.1–3.22.29. 10.1002/0471143030.cb0322s30 18228490

[jev212058-bib-0062] Théry, C. , Witwer, K. W. , Aikawa, E. , Alcaraz, M. J. , Anderson, J. D. , Andriantsitohaina, R. , Antoniou, A. , Arab, T. , Archer, F. , Atkin‐Smith, G. K. , Ayre, D. C. , Bach, J. ‐. M. , Bachurski, D. , Baharvand, H. , Balaj, L. , Baldacchino, S. , Bauer, N. N. , Baxter, A. A. , Bebawy, M. … Zuba‐Surma, E. K. (2018). Minimal Information for Studies of Extracellular Vesicles 2018 (MISEV2018): A Position Statement Of The International Society For Extracellular Vesicles And Update of the MISEV2014 guidelines. Journal of Extracellular Vesicles, 7(1), 1535750. 30637094 10.1080/20013078.2018.1535750 PMC6322352

[jev212058-bib-0063] Wiklander, O. P. B. , Nordin, J. Z. , O'loughlin, A. , Gustafsson, Y. , Corso, G. , Mäger, I. , Vader, P. , Lee, Yi , Sork, H. , Seow, Y. , Heldring, N. , Alvarez‐Erviti, L. , Smith, Ci E. , Le Blanc, K. , Macchiarini, P. , Jungebluth, P. , Wood, M. J. A. , & Andaloussi, S. E.l (2015). Extracellular vesicle in vivo biodistribution is determined by cell source, route of administration and targeting. Journal of Extracellular Vesicles 4, 26316. 25899407 10.3402/jev.v4.26316 PMC4405624

[jev212058-bib-0064] Yang, J. , Zhang, X. , Chen, X. , Wang, L. , Yang, G. (2017). Exosome mediated delivery of MiR‐124 promotes neurogenesis after ischemia. Molecular Therapy – Nucleic Acids, 7, 278–287. 28624203 10.1016/j.omtn.2017.04.010 PMC5415550

[jev212058-bib-0065] Yang, T. , Martin, P. , Fogarty, B. , Brown, A. , Schurman, K. , Phipps, R. , Yin, V. P. , Lockman, P. , & Bai, S. (2015). Exosome delivered anticancer drugs across the blood‐brain barrier for brain cancer therapy in Danio Rerio. Pharmaceutical Research, 32 (6), 2003–2014. 25609010 10.1007/s11095-014-1593-y PMC4520542

[jev212058-bib-0066] Yuan, D. , Zhao, Y. , Banks, W. A. , Bullock, K. M. , Haney, M. , Batrakova, E. , & Kabanov, A. V. (2017). Macrophage exosomes as natural nanocarriers for protein delivery to inflamed brain. Biomaterials, 142, 1–12. 28715655 10.1016/j.biomaterials.2017.07.011 PMC5603188

