# Supplementary material for: Extracellular vesicles from recombinant cell factories improve the activity and efficacy of enzymes defective in lysosomal storage disorders
Source: J Extracell Vesicles. 2021 Mar 12;10(5):e12058. doi: 10.1002/jev2.12058 (PMC7953474; doi:10.1002/jev2.12058)
Supplement: Supplementary file 1 — Supporting information. [file JEV2-10-e12058-s001.docx]

Extracellular Vesicles from Recombinant Cell Factories Improve the Activity and Efficacy of Enzymes Defective in Lysosomal Storage Disorders

Joaquin Seras-Franzoso^∞#^, Zamira V. Díaz-Riascos∞^∞#§^, José Luis Corchero^#‡^, Patricia González^∞#^, Natalia García-Aranda∞^∞#§^, Mònica Mandaña∞#§, Roger Riera∆, Ana Boullosa^∞#§^, Sandra Mancilla^∞#§^, Alba Grayston^∂^, Marc Moltó-Abad∞^¥^, Elena Garcia-Fruitós^#‡†^, Rosa Mendoza^#‡^, Guillem Pintos-Morell^∞¥^, Lorenzo Albertazzi^∆^, Anna Rosell^∂^, Josefina Casas^ƒ+^, Antonio Villaverde^#‡^, Simó Schwartz Jr^∞#^*, Ibane Abasolo^∞#§^*

SUPPORTING MATERIAL

**1. Experimental section**

*EVs isolation by Tangential Flow Filtration.* TFF method adapted from Bussatto et al (2018) was employed to isolate EV-GLA. Briefly, 100 mL of supernatant was sequentially processed in a KrosFlo Research TFF system at a TMP of 4-4.5 PSI and 53 mL/min. First, big particles were removed by filtration through 0.65 µm, using a Modified Polyethersulfone (mPES) MidiKros column. Note that this process implied the application of 4 volumes of PBS into the system diluting our initial sample 4 times. Permeate was then concentrated with a 500 KDa Polysulfone (PS) MidiKros column until 50 mL. Using the same column the sample was diafiltered with 4 additional volumes of PBS, this step allowed to further remove any EV contaminant under the 500 KDa pore cutoff, like soluble proteins. Finally, the retentate was further concentrated and EV-GLA analyzed by Western blot and enzymatic activity determination as described above. Enzymatic activities of TFF-purified samples were referred to those of precipitation-purifed (PP) samples. TFF equipment was part of the ICTS-Nanbiosis, Unit 6 (https://www.nanbiosis.es/portfolio/u6-biomaterial-processing-and-nanostructuring-unit/)

*EVs 30% sucrose cushion preparation.* EV-GLA were post- purified using the ultracentrifugation (UC) method on a 30% sucrose cushion detailed by Thery and co-workers (2016). Specifically, 15 µg of either free enzyme or EV-GLA were diluted in 11 mL PBS and carefully loaded into 13.2 mL UC tubes containing 1 mL of 30% sucrose solution. These samples were centrifuged in a Himac Ultracentrifuge CP100NX, 70 min at 100,000 *g* and 4ºC in a first UC round, UC1. Sucrose fraction from each tube was recovered and diluted in 12 mL PBS while pellets were resuspended in 50 µL PBS. Then PBS-diluted sucrose fractions were centrifuged again 70 min at 100,000 g and 4ºC in new UC tubes to obtain EV pellets in a second UC round, UC2. These pellets were also resuspended in 50 µL PBS and all the samples submitted to Western blot and enzymatic activity determination as described above. In the case of enzymatic activities, values of EV-GLA were referred to those of GLA in UC1. Triplicates were carried out. An equivalent procedure was also performed for EV-SGSH although lower amounts of EV-SGSH (5 µg) were loaded per tube.

*EVs iodixanol (*Optiprep^TM^ *) gradient characterization.* EVs were further characterized by density gradient as described by Lobb et al (2015). Briefly, Optiprep^TM^ 60% (w/v) (Sigma) was diluted in a 0.25 M sucrose, 10 mM Tris pH 7.5 solution to produce a discontinuous iodixanol/sucrose gradient comprising 40%, 20% , 10% and 5% w/v iodixanol layers. Layers were generated by the careful addition of 3 mL of each solution, with exception of 5 % solution in which case only 1.5 mL was layered, into 14 X 89 mm Ultra-Clear^TM^ centrifuge tubes (Beckman-Coulter). A total volume of 500 μL containing 750 μg of EV-GLA in PBS were loaded on top of the gradient and centrifuged for 18 h at 100,000 *g* at 4°C in a Himac Ultracentrifuge CP100NX in a swinging bucket rotor TH-641 (Sorvall). Fractions of 1 mL were collected from the top of the tube and named from F1 to F11. Recovered fractions were then diluted in PBS (10mL) and samples pelleted by centrifugation, 18 h at 100,000 *g* at 4°C. Pellets were resuspended in 150 μL of PBS and protein content and enzymatic activities determined as described above. Enzymatic activities in this case were referred to the relative GLA content determined by Western blot. Additionally, those fractions displaying either significant values of enzymatic activity or presence of GLA protein were submitted to DLS analysis. Fraction density was determined by measuring iodinaxol absorbance at 340 nm. Note that fractions were previously diluted in ultrapure water 1:1, twice. Densities values were inferred from a standard curve of known iodixanol concentrations and their respective densities.

In the case of DiR labeled EVs, labeling and subsequent dialysis of EV-GLA was performed as described above prior to loading the samples in the Optiprep density gradient. The resulting fractions were directly used to determine fluorescence emission in a IVIS Spectrum (Perkin Elmer) and further quantified using the Living Image software 4.5 (Perkin Elmer).

*DLS.* EVs hydrodynamic diameter distribution was determined by dynamic light scattering (DLS) using a NanoZS (Malvern Instruments, UK). Sample measurements were performed in triplicate at 25°C.

*Inhibition of EVs uptake routes*. 40,000 cells were seeded on 96 well plates and incubated O.N. in a humidified incubator, 37ºC, 5 % CO_2_. Then, cell media was replaced with serum-free OptiMEM containing the following chemical inhibitors: dynasore (DYN) 80 µM –inhibition of endocytic vesicle scission from cell membrane–, chlorpromazine (CHP) 20 µM –inhibition of clathrin mediated endocytosis–, nystatin (NYS) 50 µM –inhibition of caveolae mediated endocytosis– and 5-(N-ethyl-N-isopropyl) amiloride (EIPA) 100 µM –inhibition of macropynocitosis–. Plates were incubated 1 h at 37ºC, 5 % CO_2_ and medium replaced by complete fresh medium supplemented with 2,5 µg/mL of DiD labeled EV-GLA. Cells were further cultured for 2 and 4 h and finally prepared for flow cytometry analysis, as described in the main text. Cells treated with chemical inhibitors and without EV were included as viability controls monitored by DAPI staining. All conditions were tested in triplicates.

*Hemolysis and plasma aggregation assays.* Two types of assays were performed to test the hemocompatibility of the EVs. On the one hand, their effect on the integrity of red blood cells was measured using a hemolysis test. On the other hand, their potential interference with blood coagulation was studied by analyzing the plasma coagulation times.

In detail, for the hemolysis test, red blood cells (RBC) were isolated from volunteer donors, resuspended in 2% (v/v) of PBS, and exposed to different concentrations of test compounds during 1 h at 37 °C in duplicates. The amount of released hemoglobin was measured in a spectrophotometer at 405 nm (Biotek ELx800) after centrifugation (1000 g, 10 min). Absorbance values were referred to a positive control of 100% hemolysis obtained after incubating RBC with 1% of Triton-X. Samples with <5% are considered non hemolytic.

The effect of the vesicles in plasma coagulation was tested using Start4 equipment (Stago, France) and following the manufacturer’s protocol to determine the prothrombin time (PT).

*Gb3 and LysoGb3 determinations.* Gb3 and LysoGb3 levels were determined with LC-HRMS at the Institute of Advanced Chemistry of Catalonia (IQAC-CSIC). In detail, 750 µL of a methanol-chloroform (2:1, vol/vol) solution containing internal standards (N -dodecanoylsphingosine, N -dodecanoylglucosylsphingosine, N -dodecanoylsphingosylphosphorylcholine, and N –heptadecanoylceramide trihexoside, 0.2 nmol each) were added to plasma (0.1 mL) or kidney homogenates (0.1 mL, around 0.3 mg/mL protein). Samples were extracted at 48°C overnight and cooled, 75 µl of 1M KOH in methanol was added, and the mixture was incubated for 2 h at 37°C. Following addition of 75 µL of 1M acetic acid, samples were evaporated to dryness and stored at -20°C until the analysis of sphingolipids. Before the analysis 150 µL of methanol were add to the samples, centrifuged at 13000 g for 5 min and 130 µL of the supernatant were transferred to a new vial and injected. Sphingolipids were measured with an Acquity ultraperformance liquid chromatography (UPLC) system connected to a time-of-flight (LCT Premier XE) detector controlled with Waters/Micromass MassLynx software. Sample was injected onto an UPLC BEH C8 column (particle size, 1.7 µm; 100 mm by 2.1 mm); flow rate of 0.3 ml/min and column temperature of 30°C were used. The mobile phase was methanol with 1 mM ammonium formate and 0.2% formic acid (solution A)–water with 2 mM ammonium formate and 0.2% formic acid (solution B). Gradient elution started at 80% solution A, was increased to 90% solution A over 3 min, held for 3 min, increased to 99% solution A over 9 min, and after held for 3 min. Initial conditions were attained for 2 min, and the system was stabilized for 3 min. The acquisition range of the TOF detector was m/z 50 to 1500, the capillary voltage was set to 3.0 kV, the desolvation temperature was 350°C, and the desolvation gas flow rate was 600 liters/h. Quantification was carried out using the ion chromatogram obtained for each compound using 50 mDa windows. The linear dynamic range was determined by injection of standard mixtures. Positive identification of compounds was based on accurate mass measurements with an error <5 ppm and LC retention time, compared to that of a standard (<2%). Quantification of was carried out against internal standard (N –heptadecanoylceramide trihexoside) whereas lysoGb3 was quantified using external standard calibration.

**2. Results**


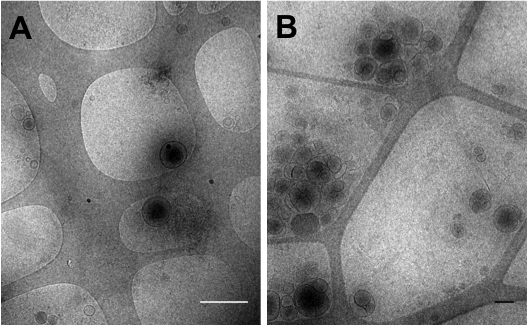


**Figure 1 S. Low magnification TEM images of lysosomal enzyme loaded EVs. A)** EV-GLA. **B)** EV-SGSH. Magnification bar corresponds to 200 nm in both images.


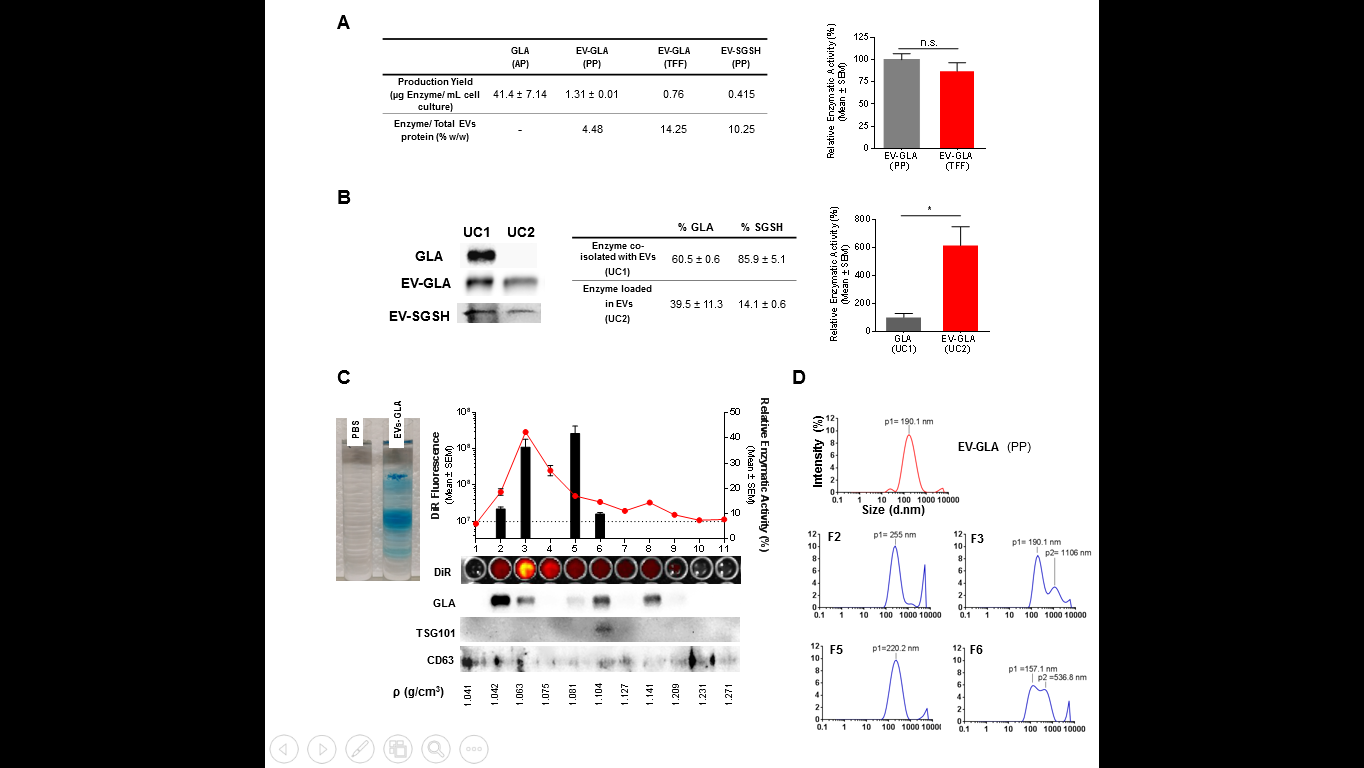


**Figure S2. Characterization of recombinat enzyme production in EVs. A)** Production yields and recombinant protein content by affinity purification (AP) precipitation method (PP) or tangential flow filtration (TFF) and their specific enzymatic activity, as referred to that seen in EV-GLA purified by PP. **B)** Sucrose cushion purification of GLA and SGSH containing EVs: protein detection by Western blot in the first and second round of ultracentrifugation (UC1 and UC2, respectively) and their relative enzymatic activity. Soluble free GLA was included as control of the UC process, and enzymatic activities were referred to those found for free GLA in UC1. **C)** Characterization of different fractions (F1 to F11) obtained from iodixanol gradient purification**,** including, DiR fluorescence (red line corresponds to specific signal in each fraction, dotted line the background signal), relative enzymatic activity (black columns, as referred to total specific enzymatic activity), GLA content, TSG101 and CD63 expression by Western blot. Densities corresponding to each iodixanol fraction are displayed below. **D**) Mean size of EVs by dynamic light scattering in different iodixanol fractions.

Iodixanol density gradient separation allowed us to obtain a better resolution of the nanoparticles present within the samples isolated from cell supernatants. The protein of interest was detected at significant levels (**Figure S2C**) at low density fractions, lower than 1.06 g/cm^3^, but also at densities corresponding to extracellular vesicles fractions between 1.08 – 1.14 g/cm^3^. Low density fractions, mainly F3, showed a much higher fluorescence than the rest. This fact indicated a significantly stronger interaction of DiR with a lipid environment, suggesting the presence of Low Density Lipoprotein (LDL) particles. LDL particles due to their hydrophobic core are much more prone to be inspecifically labeled by hydrophobic dyes like DiR. Besides, the presence of these lipid structures is in agreement with previous reports showing LDL particles at similar buoyancies (Karimi et al 2018). However, when looking at the size distribution of this particular fraction bigger particles than expected were observed, including a significant peak with a mean diameter of 1.1 μm. This result suggests that part of such lipid material could correspond to fragments of membrane from damaged EVs. Of note, DLS distribution of particle size by intensity tends to render bigger values than the particles measured by NTA or TEM. This phenomenon is due to the presence of bigger particles within the samples, much more intense than the smaller ones although being only a minimal percentage in number. The detection of CD63, tetraspanin associated to EVs membrane, in F1, F2, F3 fractions (Figures S2C) supports this latter hypothesis.

Concomitantly, the intense GLA band observed in F2 coupled with its lower enzymatic activity with respect to F3 suggested that free GLA, previously shown less active than when inside vesicles or liposomes (Cabrera et al. 2016), could be attached to lipoprotein particles. This fact is supported by the previous quantification of free enzyme by TFF and sucrose cushion purification. In those experiments the free GLA fraction was estimated to be around the 60%. Nevertheless, after iodixanol density gradient separation no detectable amounts of GLA were present in either the pellet or the fractions with density values higher than 1.2, where soluble protein was to be expected. Given that the starting material, cell culture supernatants of CHO cells, was the same for all three cases it can be assumed that the co-purified free enzyme was being dragged along the density gradient by another element. Since F2 was the one displaying the higher GLA protein content but not maximal levels of enzymatic activity, probably the LDL were responsible for “dragging” phenomenon. Significantly, this effect would be probably enhanced by the high concentration of GLA within the samples submitted to iodinaxol density gradient separation (750 µg of EVs in 500 µL) in comparison to TFF or sucrose cushion purified samples, that were at least 1 order of magnitude more diluted.

Regarding the fractions within the range of EVs buoyancies (1.08 – 1.14 g/cm^3^), F5, F6 and F8, exhibited relevant amounts of enzyme. From these, F5, with the lowest density, showed a higher enzymatic activity followed by the slightly denser vesicles isolated from F6. Interestingly, those particles isolated from F8 retained not detectable activity. Further, when looking at the size distribution of these samples a single peak was obtained for F5 while 2 different subpopulations with equivalent densities but different size were isolated in F6. F8 rendered DLS data with poor quality and was therefore excluded from the analysis. These results depict the co-existence of multiple types of EV regarding their density and size and are in accordance with previous reports (Brobie et al. 2012). In our particular setup, the therapeutic enzyme seem that was successfully vehiculized in particles with diverse sizes and buoyancies generating a unique product with higher efficacy in vitro and in vivo than their free counterpart.


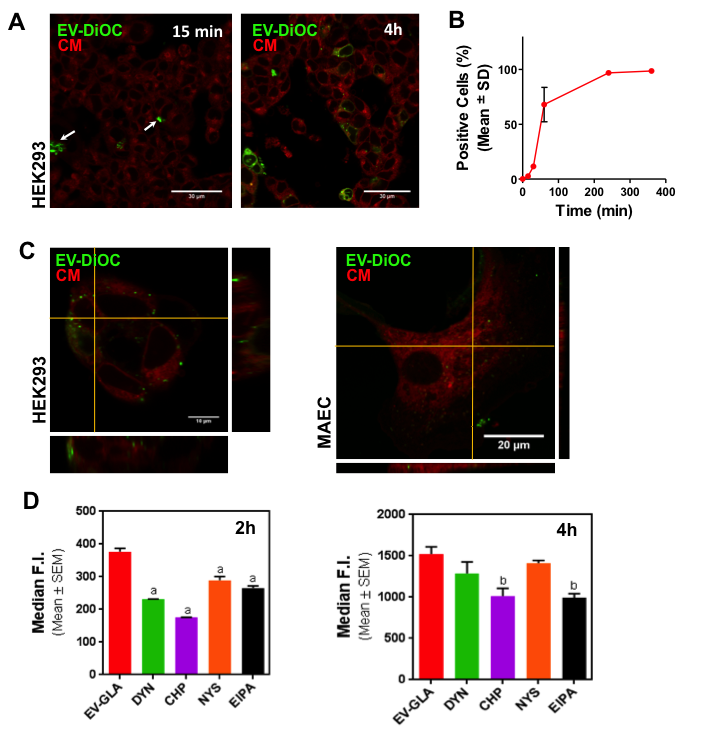


**Figure S3.** **Internalization of EV-GLA.** **A)** Confocal images of EV-GLA cell internalization in HEK293 cells at 15 min and 4 h of incubation. EVs were labeled with DiOC (green) and cell membranes were labeled with Cell Mask (red). **B)** Time course internalization of DiD-labeled EV-GLA by flow cytometry in HEK293 cells. **C)** Z-stack projection of confocal images of HEK293 cells and MAEC incubated 4 h with DiOC-labeled EV-GLA. Lateral projections show the intracellular localization of fluorescently EVs (yellow lines indicate location of projections on the right and bottom sides of the main image). EVs were labeled with DiOC (green) and cell membranes were labeled with Cell Mask (red). **D)** Influence of specific endocytosis chemical inhibitors in EVs uptake by HEK293 cells after 2 and 4 h of incubation (left and right, respectively).


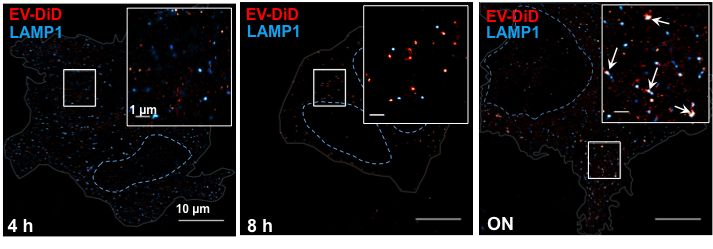


**Figure S4. STORM images of EV colocalization with LAMP1 lysosomal marker.** HEK293 cells after 4 h, 8 h and ON incubation with DiD-labeled EV-GLA. Nuclei in each cell are outlined with a blue dashed line. Insets show a cytoplasmatic region for further detail of EV-GLA and LAMP1 interaction.


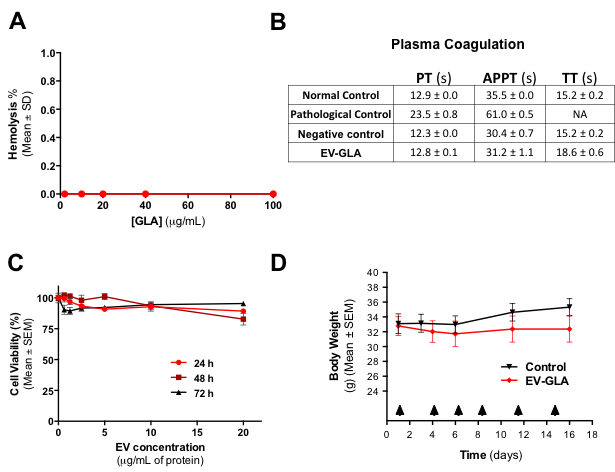


**Figure S5. Safety and tolerability of EVs.** **A)** Hemolysis assay: stability and integrity of red blood cells was not affected after 1 h incubation with different doses of EV-GLA. **B)** Plasma coagulation times (PT, pro-thrombin time; APPT, activated partial thromboplastin time and TT, thrombin time) was not affected after incubation of EV-GLA at 2.8 μg/mL of EV. **C)** Cell viability assays at 24, 48 and 72 h with HEK293 cells showing no toxicity upon incubation with different doses of EV-GLA. **D)** Animal body weight after repeated administration of EV-GLA (arrows) at 1 mg/kg of GLA.


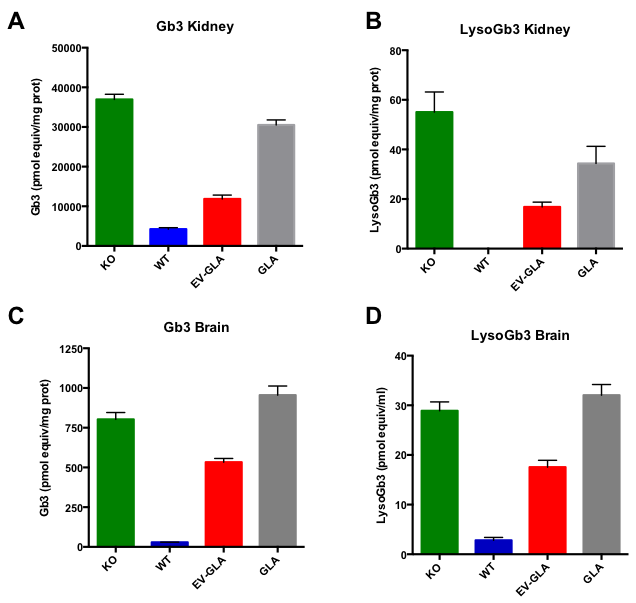


**Figure S6. Gb3 and LysoGb3 determinations in kidneys and brain parenchyma of Fabry KO mice treated intravenously with EV-GLA (1 mg/kg of GLA). A)** Gb3 levels in kidneys **B)** LysoGb3 levels in kidneys, **C**) Gb3 levels in brain parenchyma and **D**) Gb levels in brain parenchyma by LC-HRMS.

**3. References**

Bobrie, A.; Colombo, M.; Krumeich, S.; Raposo, G.; Théry, C. Diverse Subpopulations of Vesicles Secreted by Different Intracellular Mechanisms Are Present in Exosome Preparations Obtained by Differential Ultracentrifugation. J. Extracell. Vesicles 2012, 1. <https://doi.org/10.3402/jev.v1i0.18397>.

Busatto, S.; Vilanilam, G.; Ticer, T.; Lin, W.-L.; Dickson, D.; Shapiro, S.; Bergese, P.; Wolfram, J. Tangential Flow Filtration for Highly Efficient Concentration of Extracellular Vesicles from Large Volumes of Fluid. *Cells* **2018**, *7* (12), 273. <https://doi.org/10.3390/cells7120273>.

Cabrera, I.; Abasolo, I.; Corchero, J. L.; Elizondo, E.; Gil, P. R.; Moreno, E.; Faraudo, J.; Sala, S.; Bueno, D.; González-Mira, E.; et al. α-Galactosidase-A Loaded-Nanoliposomes with Enhanced Enzymatic Activity and Intracellular Penetration. *Adv. Healthc. Mater.* **2016**, *5* (7), 829–840. https://doi.org/10.1002/adhm.201500746

Lobb, R. J.; Becker, M.; Wen, S. W.; Wong, C. S. F.; Wiegmans, A. P.; Leimgruber, A.; Möller, A. Optimized Exosome Isolation Protocol for Cell Culture Supernatant and Human Plasma. *J. Extracell. Vesicles* **2015**, *4* (1), 27031. https://doi.org/10.3402/jev.v4.27031

Théry, C.; Amigorena, S.; Raposo, G.; Clayton, A. Isolation and Characterization of Exosomes from Cell Culture Supernatants and Biological Fluids. *Curr. Protoc. Cell Biol.* **2006**, *30* (1), 3.22.1-3.22.29. https://doi.org/10.1002/0471143030.cb0322s30.
